# Inhibition of Rho GEFs attenuates pulmonary fibrosis through suppressing myofibroblast activation and reprogramming profibrotic macrophages

**DOI:** 10.1038/s41419-025-07573-5

**Published:** 2025-04-11

**Authors:** Chengju Luo, Chenqi Huang, Yuqi Zhu, Yuxin Zhou, Yansheng Qiao, Chenxiao Shi, Yuan Gao, Yongjian Guo, Libin Wei

**Affiliations:** 1 Jiangsu Key Laboratory of Carcinogenesis and Intervention, China Pharmaceutical University, #24 Tongjiaxiang, Nanjing, 210009 China; 2 Department of Pharmacy, Affiliated Hospital of Nantong University, Nantong, 226001 China; 3 Bayi Hospital Affiliated to Nanjing University of Chinese Medicine, #138 Xianlin Rd, Nanjing, 210023 China; 4 Public Laboratory Platform, China Pharmaceutical University, #24 Tongjiaxiang, Nanjing, 210009 China; 5 School of Biopharmacy, China Pharmaceutical University, #639 Longmian Avenue, Nanjing, 211198 China

**Keywords:** Respiratory tract diseases, Pharmacology, Target identification

## Abstract

Idiopathic pulmonary fibrosis has a poor prognosis, with existing medications only partially alleviating symptoms, highlighting the urgent need for new therapeutic approaches. The dysregulations of Rho GTPases/ROCK are related with various diseases, including fibrosis. Nevertheless, the development of drugs for pulmonary fibrosis treatment has predominantly concentrated on ROCK inhibitors. Small GTPases have been historically recognized as “undruggable”. Here, we explore a novel Rho GEFs inhibitor GL-V9, and find that GL-V9 alleviates bleomycin-induced pulmonary fibrosis in mice by inhibiting myofibroblast activation and reprogramming profibrotic macrophages. Distinct from the mechanisms of the first-line drug Nintedanib, GL-V9 binds to the DH/PH domain of Rho GEFs and block the activation of Rho GTPase signaling. This action subsequently suppresses myofibroblast activation by interfering with Rho GTPase-dependent cytoskeletal reorganization and the activity of MRTF and YAP, and inhibits M2 macrophage polarization by modulating RhoA/STAT3 activity. The discovery of new regulatory mechanisms of GL-V9 suggests that targeting Rho GEFs represents a potent strategy for pulmonary fibrosis treatment.

## Introduction

Idiopathic Pulmonary Fibrosis (IPF) is an interstitial lung disease of an indeterminate cause that primarily manifests as an excessive accumulation of extracellular matrix (ECM), resulting in the remodeling of lung tissue and irreversible impairment of pulmonary function [1]. The incidence of idiopathic pulmonary fibrosis is approximately 3–9 cases per 100,000 people per year, comparable to the incidence of malignant tumors such as gastric cancer and liver cancer [2]. The prognosis for IPF is poor, with patients having a median survival period of 3–5 years from diagnosis [3]. The majority of patients are diagnosed in the late stages of the disease, which results in a limited range of treatment options. Currently, the U.S. Food and Drug Administration (FDA) has approved two drugs for IPF treatment: Nintedanib, a small molecule inhibitor of tyrosine kinases that impedes the activation, growth, and dispersal of fibroblasts, and Pirfenidone, a pyridone derivative noted for its antifibrotic, anti-inflammatory, and antioxidative effects. These drugs have been demonstrated to significantly decelerate the decline in forced vital capacity (FVC), yet a considerable number of patients continue to experience disease progression to more severe stages despite treatment [4, 5].

IPF arises from an impaired regeneration of alveolar epithelial cells and a dysregulated wound-healing response [6]. Factors such as genetic predispositions, environmental exposures, and age-related physiological changes lead to sustained or repeated damage to epithelial cells. This damage triggers abnormal activation and inflammatory reactions in these cells, processes that are primarily mediated by pro-inflammatory cytokines like Transforming growth factor-β (TGF-β). Such cytokines drive the migration, proliferation, and differentiation of fibroblasts into myofibroblasts, which play a central role in fibrosis. Myofibroblasts synthesize and secrete substantial quantities of extracellular matrix (ECM) components, including collagens, glycoproteins, and proteoglycans, which promote the formation of fibrous scar tissue. Moreover, enhanced cross-linking of collagen provides resistance to proteolytic degradation, resulting in irreversible scarring and structural damage to the tissue [7]. Myofibroblasts have a developed cytoskeletal structure containing actin and myosin, which are connected to mature focal adhesions, linking the cytoskeleton to the ECM [8]. This enables myofibroblasts to contract the surrounding ECM and produce contracture, thereby impeding organ function. The expression of α-smooth muscle actin (α-SMA) in myofibroblasts further enhances the contractile ability of smooth muscle cells, directly leading to several critical features of pulmonary fibrosis (PF), including ECM protein deposition, tissue scar formation, and lung tissue remodeling [9, 10].

RhoA, a member of the Rho GTPase family, plays a crucial role in regulating various cellular processes, including cytoskeletal rearrangement, cell motility, and cell polarity [11]. The activation of the Rho GTPase family is dependent on the action of Rho guanine nucleotide exchange factors (Rho GEFs), which facilitate the exchange of GDP for GTP on Rho proteins [12]. RhoA, mainly through its major effector proteins Rho-associated protein kinase (ROCK) and mammalian diaphanous related formin 1 (mDia1), is responsible for orchestrating cytoskeletal dynamics. ROCK phosphorylates multiple downstream targets to regulate the stability of F-actin, while mDia1 is thought to play a role in the nucleation of F-actin [13]. The reorganization of the actin cytoskeleton is critical in both normal wound healing and the fibrotic process in pathological states, including the recruitment of fibroblasts, differentiation of myofibroblasts, and re-epithelialization [14, 15]. Moreover, changes in the actin cytoskeleton also affect the subcellular localization of transcriptional co-factors, which in turn modulates the expression of genes associated with fibrosis [16].

Emerging evidence indicates that the recurrent micro-injuries sustained by alveolar epithelial cells in IPF triggers the aberrant activation of immune cells [17], including macrophages [18], neutrophils, T lymphocytes [19], and mast cells. The subsequent release of various cytokines and chemokines facilitates the proliferation of stromal cells, which in turn induces epithelial-mesenchymal transition (EMT) and promotes the excessive proliferation of fibroblasts and myofibroblasts, leading to significant structural remodeling of lung tissue. The investigation into the macrophage plasticity in relation to the advancement of PF is the most comprehensive. The correlation between ROCK activity and macrophage polarization is also increasingly recognized [20, 21]. Macrophages, which are highly plastic and multifunctional cells, can differentiate into either classically activated M1 macrophages or alternatively activated M2 macrophages. During the wound healing phase, M2 macrophages are more actively engaged, possessing robust phagocytic capabilities and producing anti-inflammatory cytokines, including TGF-β, IL-10, and CTGF. These cytokines help mitigate inflammation and promote tissue repair. However, cytokines secreted by M2 macrophages also have the potential to promote fibrosis [22–24]. In patients with pulmonary fibrosis, an imbalance in the polarization ratio between M1 and M2 macrophages is observed, with a persistent increase in M2 macrophages leading to the excessive release of pro-fibrotic mediators, thus inducing the activation of myofibroblasts and excessive accumulation of collagen.

GL-V9, a newly synthesized flavonoid derived from wogonin [25], has been demonstrated to possess therapeutic potential in the treatment of fibrosis. Research in the field of liver fibrosis has demonstrated that GL-V9 can impede the progression of this condition by either inhibiting the TGF-β/Smad signaling pathway [26] or by upregulating GATA4 [27], which induces senescence in hepatic stellate cells. Additionally, GL-V9 and homologous compounds, such as wogonin, have been shown to affect the cytoskeleton [28] and cell polarity [29] in cancer cells. This study builds upon the prior research to investigate the potential role and underlying mechanisms of GL-V9 in the treatment of PF.

## Materials and methods

### Reagents

GL-V9 (C_24_H_27_NO_5_, (5-hydroxy-8-methoxy-2-phenyl-7-(4-(pyrrolidine-1-yl) butoxy) 4H-chromen-4-one), 98% purity) was synthesized and provided by Prof. Zhiyu Li [30]. Bleomycin sulfate (cat# HY-17565), Nintedanib (cat# HY-50904), and the STAT3 inhibitor C188-9 (cat# HY-112288) were purchased from MCE (NJ, USA). Oleoyl lysophosphatidic acid (LPA, cat# 857130P) were purchased from Merck Ltd (Beijing, China). Recombinant Mouse TGF-β1 (cat# CK33) and Mouse IL-4 (cat# CK15) were purchased from Novoprotein (Shanghai, China). Antibodies against RhoA (cat# ab187027), p-YAP1(cat# ab76252), p-LATS1 (cat# ab305029), p-STAT3 (cat# ab267373), and p-STAT6 (cat# ab263947) were purchased from Abcam (Cambridge, UK).Antibodies against β-actin (AC026), HA-Tag(AE036), Vinculin(A2752), p-MLC-T18/S19 (AP0955), Lamin A/C(A0249), and β-Tubulin (AC015) were purchased from Abclonal (Wuhan, China). Antibodies against GST-Tag(10000-0-AP), MLC (15354-1-AP), Collagen I (66761-1-Ig), Fibronectin (15613-1-AP), α-SMA (67735-1-Ig), YAP1 (13584-1-AP), TAZ (23306-1-AP), CD206 (60143-1-Ig), and Arg1 (16001-1-AP) were purchased from Proteintech (Wuhan, China). Alexa Fluor 488 and 546 conjugated secondary antibodies were purchased from Invitrogen (California, USA). Transforming Growth Factor Beta 1 ELISA Kit (RK00055) was purchased from Abclonal (Wuhan, China).

### Animal experiments

The Institutional Animal Care and Use Committee of China Pharmaceutical University approved animal studies. The female C57BL/6J mice in 6- to 8-week-old were anaesthetised via intraperitoneal injection of 1.25% tribromoethanol (Nanjing Aibei Biotechnology, China) at a dose of 0.2 mL/20 g. The pulmonary fibrosis model was established in C57BL/6J mice through transoral instillation of bleomycin at a dose of 2U/kg, following protocols outlined in previous studies [31]. It was reported that female mice exhibit a more consistent response to bleomycin [32]. Mice were randomly divided into four groups: the control group (Control), the bleomycin-induced pulmonary fibrosis model group (BLM), the group treated with GL-V9 immediately after bleomycin administration (BLM + GL-V9), and the group treated with GL-V9 starting on the seventh-day post-bleomycin administration (BLM + GL-V9 (DAY7)). GL-V9 was administered at a dose of 300 mg/kg using an every-other-day oral gavage regimen. Lung specimens and BALF were harvested on days 7, 14, and 21 after the establishment of the model. To compare the effect of GL-V9 and Nintedanib in vivo, bleomycin-induced pulmonary fibrotic mice were treated with GL-V9 (i.g., 300 mg/kg, once every day) or Nintedanib (i.g., 60 mg/kg, once every day), respectively, starting on day 7 after bleomycin administration.

For the mechanism investigation in vivo, mice were randomly divided into five groups: the control group (Control), the bleomycin-induced pulmonary fibrosis model group (BLM), the group co-treated with LPA and bleomycin (BLM + LPA), the groups treated with GL-V9 immediately after bleomycin administration (BLM + GL-V9), or after the combined administration of bleomycin and LPA (BLM + LPA + GL-V9). GL-V9 was administered at a dose of 300 mg/kg using an every-other-day oral gavage regimen. LPA was administered at a dose of 1.5 mg/kg every day by intraperitoneal injection. Lung specimens were harvested on days 15 after the establishment of the model.

We have now included the sample sizes in the relevant sections of the manuscript. In the survival curve experiments, each group comprised 15 mice. In the experiments designed to assess changes in body weight, the number of groups varied according to the requirements for subsequent sample collection and the results of the survival curves. We were not blinded to the group allocation during the experiment and/or when assessing the outcome.

### Cell culture and treatment

NIH/3T3 (CSTR:19375.09.3101MOUSCSP515), NCTC clone 929 (L929, CSTR:19375.09.3101MOUSCSP5039), and RAW 264.7 (CSTR:19375.09.3101MOUSCSP5036) cell lines were obtained from the Nation Collection of Authenticated Cell Cultures (Shanghai, China). Primary mouse lung fibroblasts were extracted from the lung tissues of 6-week-old female C57BL/6J following protocols outlined in previous studies [33]. Bone Marrow–Derived Macrophages (BMDMs) were extracted from the leg bones of 6-week-old female C57BL/6J. BMDMs were cultured in DMEM supplemented with 30% L929-conditioned medium and 10% FBS, following protocols outlined in previous studies [34]. Primary mouse lung fibroblasts were induced into myofibroblasts by 10 ng/mL TGF-β1 treatment after 24 h of serum-free starvation with the addition of GL-V9 treatment. NIH/3T3 cells induced into myofibroblasts using 10 ng/mL TGF-β1 treatment in serum-free DMEM medium with the addition of GL-V9 treatment. BMDMs and RAW264.7 induced M2 macrophage polarization using 20 ng/mL IL-4 treatment with the addition of GL-V9 treatment.

### Histopathology and immunohistochemical staining

Histopathology staining was performed by AiFang Biological (Hunan, China). Tissues were fixed in 4% paraformaldehyde and embedded in paraffin to prepare tissue sections. Hematoxylin and eosin (H&E) and Masson’s trichrome staining were carried out according to standard protocols, and images were captured using a NanoZoomer S200 Digital slide scanner (Hamamatsu, Japan). The fibrosis progression in mouse lung tissues stained with H&E was assessed using the Ashcroft scoring system [35], with three independent pathologists evaluating and scoring the samples. Immunohistochemical staining was performed using the IHC Detect Kit from Proteintech (Wuhan, China) according to the manufacturer’s instructions. Images were obtained with either a NanoZoomer S200 Digital slide scanner or a Scope.A1 microscope (Zeiss, Germany).

### Hydroxyproline determination

The collagen content in mouse lung tissues was determined using a hydroxyproline assay kit (Nanjing Jiancheng Bioengineering Institute, China) via the alkaline hydrolysis method. Approximately 50 mg of chopped lung tissue was weighed to record the wet weight accurately. Following alkaline hydrolysis, the pH was adjusted to 6.0–6.8, and the solution was brought to the appropriate volume. The supernatant was collected for analysis after decolorization. A standard curve was constructed using the absorbance values of the standard samples, and the hydroxyproline content was calculated per milligram of wet lung tissue weight.

### Immunofluorescence and phalloidin staining

Cells were cultured in six-well plates containing coverslips. After drug treatment, the cells were fixed with 4% paraformaldehyde solution and permeabilized with 0.5% TBST buffer. Blocking was performed with 1% BSA solution. For immunofluorescence staining, primary antibodies were incubated with the cells overnight at 4 °C, followed by incubation with fluorescent secondary antibodies at room temperature for 1 h. For phalloidin staining, FITC-labeled phalloidin working solution was prepared with 1% BSA and incubated with the cells at room temperature for 1 h. Coverslips were mounted on slides using an anti-fade reagent containing DAPI. Images were captured using a STELLARIS 5 Confocal Microscope (Leica, Germany).

### RNA-sequencing

RNA sequencing and raw read processing were performed by Gene Denovo (China). RNA was extracted from cell samples, subjected to quality control, and used for library preparation. Sequencing was conducted on the Illumina NovaSeq 6000 system (USA). The raw reads underwent quality control and alignment to the reference genome, followed by quantification of gene expression levels. Gene ranking was carried out using the Signal2Noise metric in GSEA software. Enrichment scores (ES) were calculated for M2: curated gene sets and M5: ontology gene sets, with significance determined accordingly.

### Detection of RhoA and P115 binding

The BL21 (DE3) strain carrying the pLV3-GST-RhoA plasmid was used to express GST-RhoA recombinant protein, which was subsequently captured using GST purification beads. NIH/3T3 cells were transfected with the pCMV-HA-P115 RhoGEF plasmid using the jetPRIME transfection reagent (Polyplus, France) to express the HA-P115 RhoGEF recombinant protein. The collected cells were resuspended in PBS containing protease inhibitors and lysed by three freeze-thaw cycles using liquid nitrogen. The supernatant was collected by centrifugation. The beads bound with GST-RhoA recombinant protein were incubated with the cell supernatant containing HA-P115 RhoGEF, along with 50 μM or 100 μM of GL-V9, at 4 °C for 1 h with gentle mixing. The beads were collected, and the effect of GL-V9 on the binding of RhoA and P115 RhoGEF was analyzed by Western blotting.

### Detection of RhoA and DH/PH domain binding

NIH/3T3 cells were transfected with lentivirus encoding GST-ARHGEF12(765-1138aa) or GST-ARHGEF12(765-1138aa)-922A and selected with puromycin to establish stable cell lines. Additionally, NIH/3T3 cells were transfected with lentivirus encoding HA-RhoA and selected with G418 to obtain stable cell lines expressing HA-RhoA. Cells expressing both recombinant proteins were treated with 50 μM or 100 μM of GL-V9 for 3 h. The cells were then collected and lysed using NP-40 lysis buffer containing protease inhibitors. The supernatant was collected by centrifugation. GST beads were used to capture the proteins, and the effect of GL-V9 on the binding of RhoA and the DH/PH domain was analyzed by Western blotting.

### Detection of active RhoA-GTP

The BL21 (DE3) strain carrying the pGEX-2T-GST-RBD plasmid (Addgene, #15247) was used to express the GST-RBD recombinant protein, which was then captured and purified using GST purification beads. The experiment followed a previously reported protocol [36]. After drug treatment, cells were lysed in Rho kinase lysis buffer containing protease inhibitors (GlpBio, USA), and the supernatant was collected by centrifugation. The beads bound with GST-RBD recombinant protein were incubated with the cell lysate supernatant at 4 °C for 2 h with gentle mixing. The beads were collected, and the amount of captured active RhoA-GTP was analyzed by Western blotting. Additionally, after drug treatment, the distribution of active RhoA-GTP in cells was detected by immunofluorescence using GST-RBD recombinant protein and anti-GST antibody.

### Förster resonance energy transfer (FRET) detection of RhoA activity

Primary mouse fibroblasts were transfected with the RhoA2G biosensor (Addgene, #40179) using lentivirus. After drug treatment, real-time FRET imaging was performed using a STELLARIS 5 Confocal Microscope (Leica, Germany). Cells were excited with a 405 nm laser, and emissions were collected at 480/40 nm (donor channel) and 535/30 nm (FRET channel). Image quantification was conducted using LAS X software.

### Dual-luciferase reporter assay

NIH/3T3 cells seeded in 6-well plates were transfected with 2 μg of 8xGTIIC-luciferase plasmid (Addgene, #34615) and 0.2 μg of pRL-TK plasmid (Promega, E2241) using jetPRIME transfection reagent (Polyplus, France). After drug treatment, Firefly luciferase and Renilla luciferase activities were measured sequentially using a Dual-Luciferase Reporter Assay Kit (Vazyme, China). The transcriptional activity of YAP/TEAD was determined based on the ratio of Firefly to Renilla luciferase activities.

### Molecular docking

Molecular docking studies of the DH/PH domain binding with GL-V9 were performed using the Molecular Operating Environment (MOE) software (Chemical Computing Group, Canada). The crystal structure of the DH/PH domain of LARG (Leukemia-associated RhoGEF) bound to RhoA (PDB ID: 1 × 86) was imported into the software, and the RhoA protein sequence was removed. The protein structure was preprocessed using the QuickPrep function, and the small molecule underwent energy minimization. Full-atom docking was conducted, and the highest-scoring docking result was selected for analysis.

### Surface plasmon resonance (SPR) analysis

The interaction between GL-V9 and the DH/PH domain of the wild-type LARG was analyzed using the Biacore S200 (Cytiva, USA) to determine the binding affinity. According to the instrument’s user guide, purified proteins were immobilized on a CM5 sensor chip using a coupling buffer at pH 4.5. GL-V9 was diluted in PBSB containing 5% DMSO to five different concentrations (0.39 μM, 0.78 μM, 1.5625 μM, 3.125 μM, 6.25 μM) and passed over the chip to generate response signals. The binding affinity (KD) was calculated using Biacore S200 Evaluation Software.

### Statistics and analysis

All experiments were repeated independently at least 3 times. Data are presented as mean ± SEM. Unpaired t-test was used for comparisons between two independent groups. The Shapiro-Wilk test was used to assess normality, while the Brown–Forsythe test was applied to evaluate the homogeneity of variances. One-way ANOVA was used for normally distributed data with equal variances, while Welch’s ANOVA or the Kruskal–Wallis test was applied for unequal variances or non-normal data. Unless otherwise noted in the figure legend, statistical comparisons were conducted using One-way ANOVA. Statistical significance was set at *p* < 0.05, with * indicating *p* < 0.05, ** indicating *p* < 0.01, *** indicating *p* < 0.001, and **** indicating *p* < 0.0001. Statistical analysis was performed using GraphPad Prism 8.0.1.

### Additional materials and methods

Additional methods are described in Supplementary Methods. Sequences of primers are reported in Supplementary Tables S1. Original data of western blot are reported as Original Data.

## Results

### GL-V9 attenuates pulmonary fibrosis in mice induced by bleomycin

**Fig. 1:**
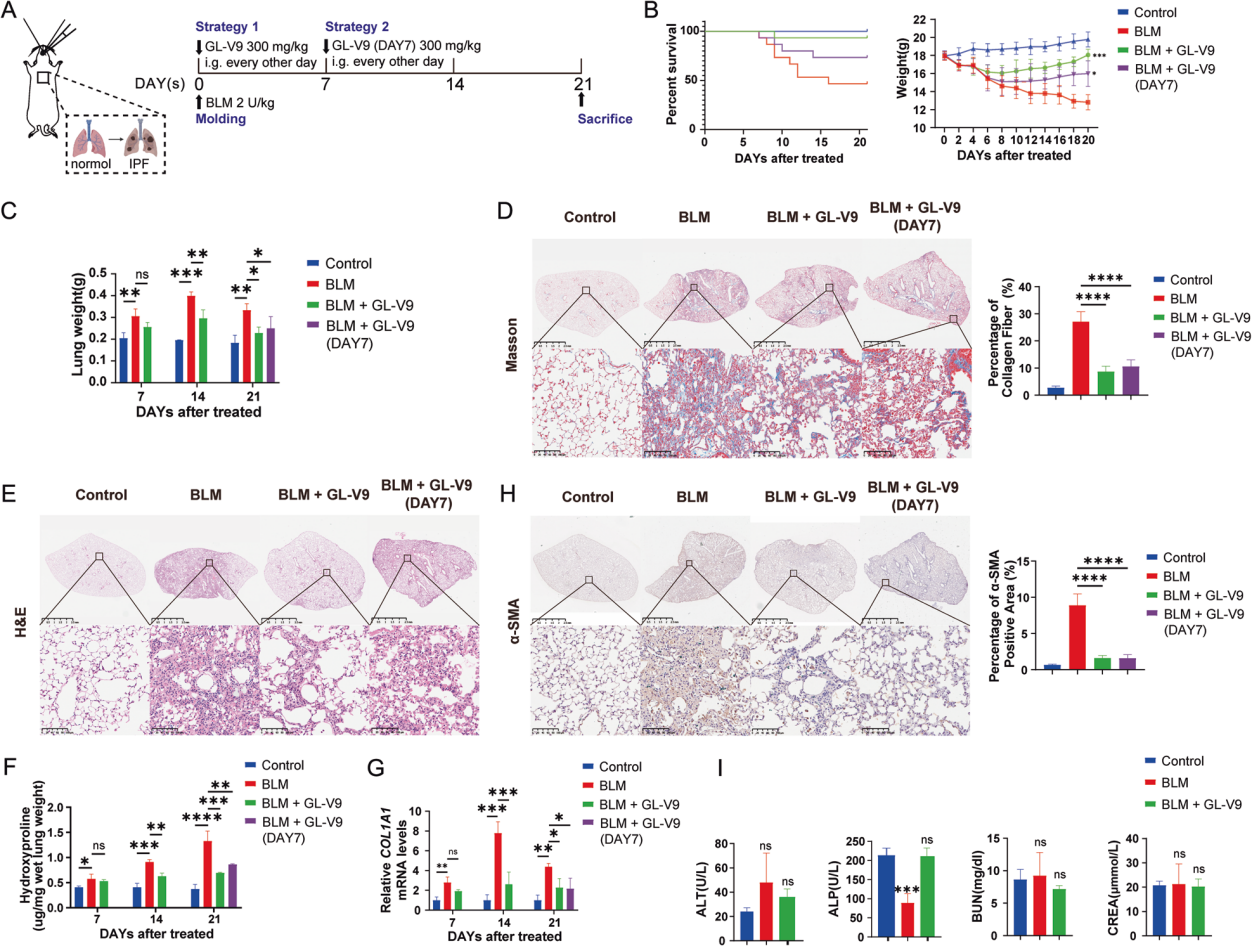
GL-V9 attenuates pulmonary fibrosis in mice induced by bleomycin. **A** Schematic representation of the experimental design for evaluating the effects of GL-V9 on bleomycin-induced pulmonary fibrosis in mice. Pulmonary fibrosis was induced by transoral instillation of bleomycin at a dose of 2 U/kg. GL-V9 was administered at a dose of 300 mg/kg on an every-other-day dosing regimen. The study was conducted in four groups: the saline control group (Control), the bleomycin-induced pulmonary fibrosis model group (BLM), the group treated with GL-V9 immediately after bleomycin administration (BLM + GL-V9), and the group treated with GL-V9 starting on day 7 after bleomycin administration (BLM + GL-V9 (DAY7)). **B** Survival curve of mice. Survival curve of mice (Each group, *n* = 15). Weight changes in mice (Control, *n* = 23; BLM, *n* = 37; BLM + GL-V9, *n* = 24; BLM + GL-V9 (DAY7), *n* = 12). **C** Lung weight changes in mice. **D** Masson stains of the left lung from mice. In Masson staining, collagen fibers appear blue-green, while the cytoplasm is stained red. The collagen fiber area in Masson-stained sections and α-SMA-positive area is quantified. **E** H&E stains of the left lung from mice. **F** Hydroxyproline content of the right lung from mice. **G** Relative *COL1A1* mRNA levels of the right lung from mice. **H** Immunohistochemical staining for α-SMA of the left lung from mice. Scale bar for the main image: 2.5 mm; enlarged section scale bars: 100 µm. **I** Serum biochemical tests for Alanine Aminotransferase (ALT), Alkaline Phosphatase (ALP), Blood Urea Nitrogen (BUN), and Creatinine (CREA) in mice. Data are shown as mean ± SEM. ns *p* > 0.05,*p < 0.05, **p < 0.01, ***p < 0.001, ****p < 0.0001.

In the pursuit of novel therapeutic approaches for IPF, a range of animal models have been developed to mimic the disease, including those induced by drugs, silica, asbestos, radiation, and genetic modifications. Of these, the bleomycin-induced model is the most extensively utilized because it replicates the histological and radiological changes seen in human IPF. To comprehensively evaluate the efficacy of GL-V9 in PF, the drug administration was initiated at two distinct time points. As shown in Fig. 1A, in Strategy1, mice were administrated with GL-V9 immediately on the day of modeling to assess its preventive effects against PF through inflammation inhibition; and in Strategy 2 mice were administrated with GL-V9 on the seventh day of modeling to determine its therapeutic potential in treating established idiopathic pulmonary fibrosis by directly targeting fibrotic processes.

The results demonstrated that GL-V9 treatment in PF mice, whether initiated during the inflammation or fibrosis phases, significantly improved survival rates and mitigated weight loss trends (Fig. 1B). Additionally, GL-V9 reduced the wet lung weight increase induced by BLM modeling (Fig. 1C). Pathological analysis of mouse lung sections using H&E staining and Masson staining revealed that GL-V9 significantly reduced the area of inflammation infiltration and fibrotic foci (Fig. 1D, E). Quantifying fibrosis using the Ashcroft score in histological samples [37] demonstrated a reduction in fibrosis in mouse lungs treated with GL-V9 (Fig. 1E). The result of Masson staining and hydroxyproline content analysis also demonstrated that GL-V9 treatment resulted in a reduction in lung collagen deposition (Fig. 1D, F). Furthermore, the expression levels of *COL1A1* mRNA were found to be significantly lower in GL-V9-treated groups, indicating a decreased rate of collagen production (Fig. 1G). Immunohistochemical staining for α-SMA was employed to quantify myofibroblasts, revealing a significant decrease in the number of lung myofibroblasts in PF mice treated with GL-V9 across both treatment strategies (Fig. 1H). Pathological examinations of major organs and biochemical tests of liver and kidney functions indicated that GL-V9 treatment did not produce significant toxic side effects in mice (Fig. 1I and Supplementary Fig. S1). These findings suggest that initiating GL-V9 treatment during either the inflammatory or fibrotic stages can effectively alleviate bleomycin-induced pulmonary fibrosis in a mouse model. Importantly, compared with the first-line drug Nintedanib, GL-V9 demonstrated comparable therapeutic efficacy in terms of survival rate, mouse body weight, and pathological examination (Supplementary Fig. S2). Thus, GL-V9 reduces the deposition of collagen and the content of myofibroblast, and significantly attenuated PF in mice.

### GL-V9 alleviates lung inflammation and inhibits M2 macrophage polarization in mice with bleomycin-induced pulmonary fibrosis

**Fig. 2:**
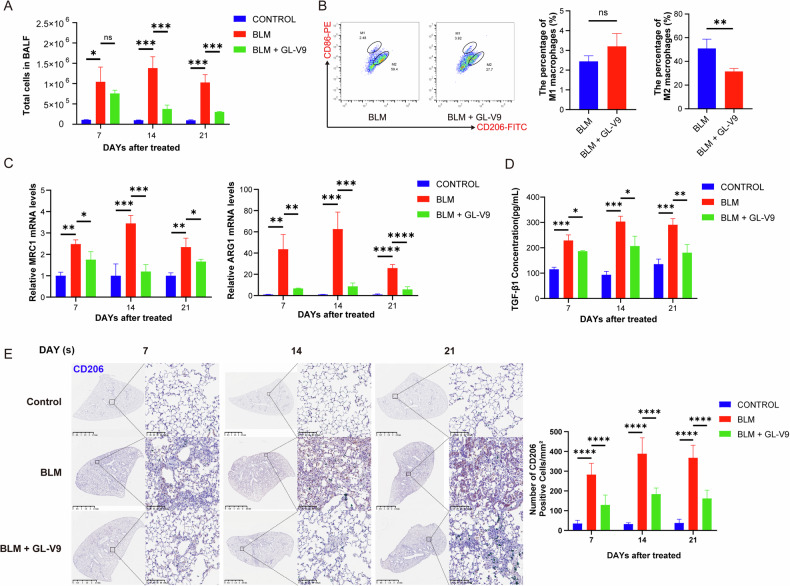
GL-V9 alleviates lung inflammation and inhibits M2 macrophage polarization in mice with bleomycin-induced pulmonary fibrosis. Mice with pulmonary fibrosis induced by transoral instillation of Bleomycin and treated with GL-V9 at 300 mg/kg on an every-other-day dosing regimen. Lung specimens and BALF were harvested on days 7, 14, and 21 after the establishment of the model. **A** Total number of cells in BALF from mice. **B** Macrophage polarization status in BALF from mice on day 7. Flow cytometry analysis of the percentage of infiltrated M2 macrophages (F4/80^+^ CD206^+^) and M1 macrophages (F4/80^+^ CD86^+^) were shown. Differences between groups were analyzed using unpaired t-test. **C** The relative mRNA levels of *MRC1* and *ARG1* in the right lung. **D** The concentration of TGF-β1 in BALF. **E** Immunohistochemical staining for CD206 in the left lung, with quantification of CD206-positive cell count. Scale bar for the main image: 2.5 mm; enlarged section scale bars: 100 µm. Data are shown as mean ± SEM. ns *p* > 0.05, *p < 0.05, **p < 0.01, ***p < 0.001, ****p < 0.0001.

The number of cells in bronchoalveolar lavage fluid (BALF) is a direct reflection of the abundance of free cells within the alveoli and smaller airways. The administration of GL-V9 resulted in a notable reduction in cell counts in BALF, indicating a decline in pulmonary inflammation in mice (Fig. 2A). Macrophage plasticity is crucial in the progression of pulmonary fibrosis. Given the significant heterogeneity among macrophages and the fibrosis-promoting effects of the M2 polarization state, flow cytometry was employed to identify polarization markers. Cells expressing F4/80^+^ CD206^+^ were classified as M2-type macrophages, whereas those expressing F4/80^+^ CD86^+^ were categorized as M1-type macrophages. The results indicated a significant decline in the proportion of M2-type macrophages in BALF following GL-V9 treatment (Fig. 2B). Then, the expression of *ARG1* and *MRC1* mRNA, genes specific to M2 macrophages, was assessed. Bleomycin treatment resulted in a significant increase in the number of macrophages, peaking on day 14. In contrast, GL-V9 administration significantly reduced the content of M2 macrophages in the lungs of PF mice (Fig. 2C). Immunohistochemical staining employing CD206 as a marker demonstrated that treatment with GL-V9 significantly attenuated the infiltration of M2 macrophages within the alveolar septa (Fig. 2E). M2 macrophages can secrete a variety of pro-fibrotic cytokines, among which excessive TGF-β1 is associated with pathological fibrosis. GL-V9 treatment resulted in a reduction in the content of TGF-β1 in BALF (Fig. 2D). These findings indicate that GL-V9 treatment leads to a decrease of M2 macrophage polarization.

### GL-V9 is a novel Rho GTPase inhibitor and disturbs the binding of RhoGEF to RhoA

**Fig. 3:**
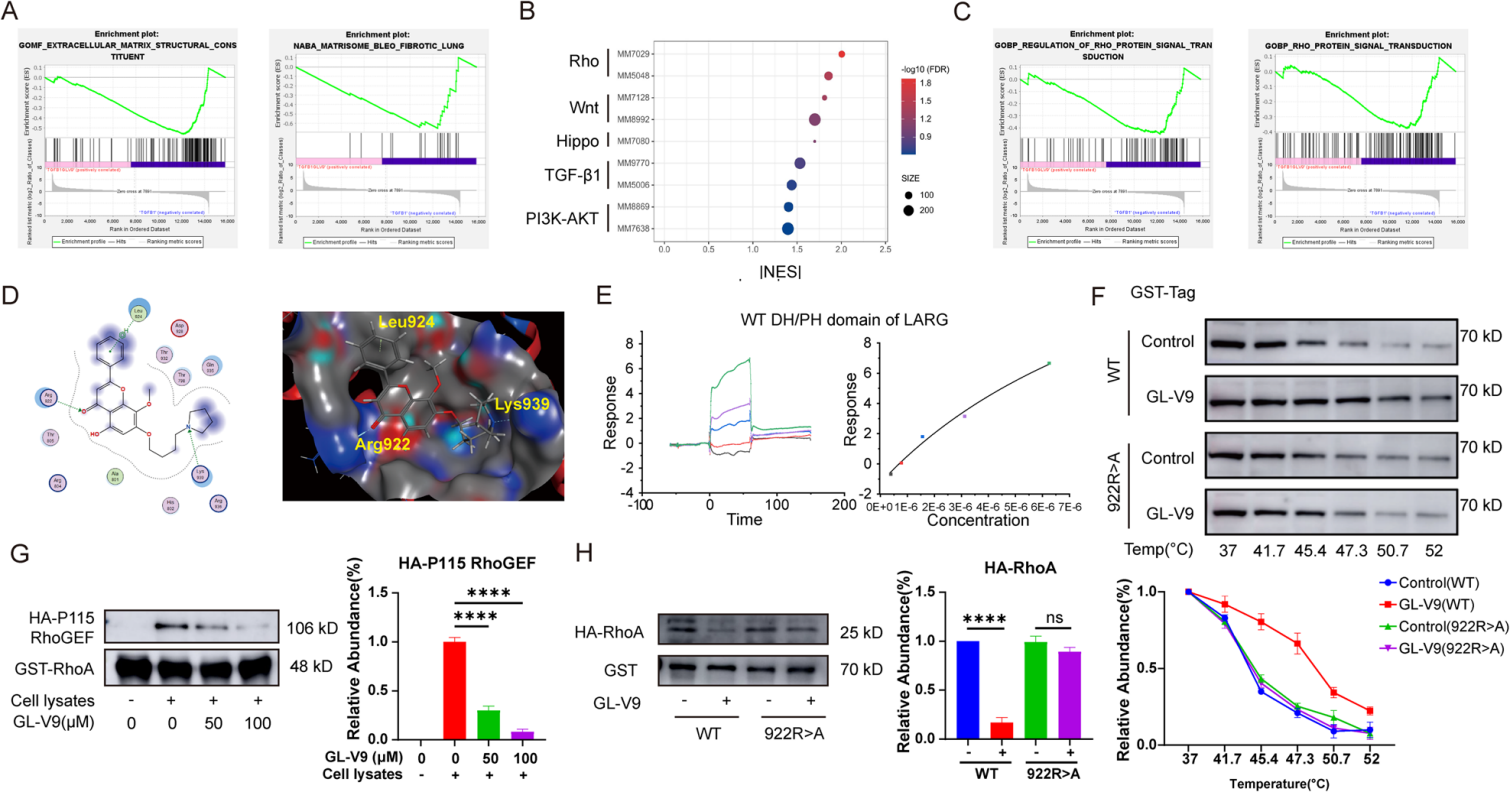
GL-V9 inhibits the binding of RhoGEF to RhoA. **A**–**C** Primary lung fibroblasts from mice were subjected to serum starvation for 24 h, followed by treatment with 5 ng/mL TGF-β1 and 7.5 μM GL-V9 for 24 h. Cells were subjected to RNA-seq analysis, and differentially expressed genes were analyzed using gene set enrichment analysis (GSEA). **A** GSEA enrichment analysis of differential genes in the GL-V9 treatment group within gene sets related to pulmonary fibrosis and extracellular matrix. The normalized enrichment score (NES) for the gene set associated with pulmonary fibrosis (MM17053, NABA MATRISOME BLEO FIBROTIC LUNG) was −2.0312, with a false discovery rate (FDR) of 0.0053. The NES for the gene set related to the extracellular matrix (M12927, GOMF EXTRACELLULAR MATRIX STRUCTURAL CONSTITUENT) was −2.7825, with an FDR of 0.0000. **B** GSEA enrichment analysis of differential genes in the GL-V9 treatment group within gene sets related to pulmonary fibroblast differentiation signaling pathways. **C** GSEA enrichment analysis of differential genes in the GL-V9 treatment group within gene sets related to Rho signaling regulation. The NES for MM7029(GOBP REGULATION OF RHO PROTEIN SIGNAL TRANSDUCTION) was −2.0048, with an FDR of 0.0134. The NES for MM5048(GOBP RHO PROTEIN SIGNAL TRANSDUCTION) was −1.8553, with an FDR of 0.0368. **D** Molecule docking examination of GL-V9 and the DH/PH domain of LARG. PDB ID: 1 × 86. **E** Surface plasmon resonance (SPR) analysis of the binding of GL-V9 to the DH/PH domain of LARG. **F** Cellular thermal shift assay (CETSA) assay for detecting the interaction between GL-V9 and the DH/PH domain of LARG in either the wild-type or ARG922 mutant form (922 R > A). NIH/3T3 cells were treated with 75 μM GL-V9 for 3 h. **G** The effect of GL-V9 on the binding between P115 RhoGEF and RhoA. **H** The effect of GL-V9 on the binding between RhoA and the DH/PH domain of LARG in either the wild-type or ARG922 mutant form. Western blot data are quantified and shown as mean ± SEM. ns *p* > 0.05, **** *p* < 0.0001.

In vivo experiments, we have preliminarily revealed that GL-V9 inhibits the activation of myofibroblasts. For the further exploration of the mechanisms, an RNA-seq study was performed to investigate the impact of GL-V9 on the transcriptome of primary mouse myofibroblasts induced by TGF-β1. The enrichment of differentially expressed genes in gene sets related to pulmonary fibrosis, as well as extracellular matrix proteins, was observed. GL-V9 significantly downregulates these genes, suggesting that GL-V9 can hinder the pulmonary fibrosis process and reduce the expression of extracellular matrix proteins (Fig. 3A). A detailed analysis of the signaling pathways that are closely associated with the onset and progression of pulmonary fibrosis revealed that GL-V9 exerted a pronounced inhibitory effect on these pathways. The Rho GTPase family gene sets exhibited the most significant inhibitory response to GL-V9 treatment (Fig. 3B, C). The activity of the Rho GTPase family depends on its binding to GTP, and Rho GEFs directly activate the Rho GTPase family by promoting the release of GDP and binding to GTP. Molecular docking and virtual screening revealed that GL-V9 forms hydrogen bonds with the ARG922, LEU924, and LYS939 residues of the DH/PH domain of LARG (Leukemia-associated RhoGEF) (Fig. 3D). The binding free energy between GL-V9 and the DH/PH domain is −7.9 kcal/mol. Notably, the LYS939 residue is located on the α5 helix of the LARG’s DH domain, while the ARG922 and LEU924 residues are situated within the α4-α5 loop of the LARG’s DH domain. The binding sites between LARG and RhoA are concentrated in the α1, α5, and α6 helices of the DH domain, and the α4-α5 loop determines the substrate specificity of LARG. The binding site for GL-V9 on the LARG’s DH/PH domain is a critical region for the interaction between LARG and RhoA. Surface Plasmon Resonance (SPR) analysis determined a binding affinity (Kd) of 11.84 μM between GL-V9 and the wild-type DH/PH domain of LARG (Fig. 3E). CETSA is a method for assessing molecular interactions by measuring the increase in protein thermal stability that occurs upon small molecule binding. GL-V9 significantly enhanced the thermal stability of the wild-type DH/PH domain, yet it failed to elicit the thermal stability of the DH/PH domain with a mutation at position ARG922 (922R>A) (Fig. 3F). LARG and its closely related homologs, P115 RhoGEF and PDZ RhoGEF, are RhoA-selective RhoGEFs. GL-V9 significantly reduced the binding affinity of HA-tagged P115 RhoGEF for the GST-tagged RhoA recombinant protein, indicating that GL-V9 effectively inhibits their interaction (Fig. 3G). GL-V9 significantly reduced the levels of HA-RhoA that is associated with the wild-type DH/PH domain with GST-tagged, but had no effect on the levels of RhoA bound to the DH/PH domain with the mutation at position ARG922 (Fig. 3H). These findings suggest that GL-V9 impedes the binding of Rho GEFs or their DH/PH domain to RhoA. However, this function may be lost following mutation at the ARG922 site of the DH/PH domain.

### GL-V9 inhibits RhoA activation of myofibroblasts and impedes the reorganization of the actin cytoskeleton

**Fig. 4:**
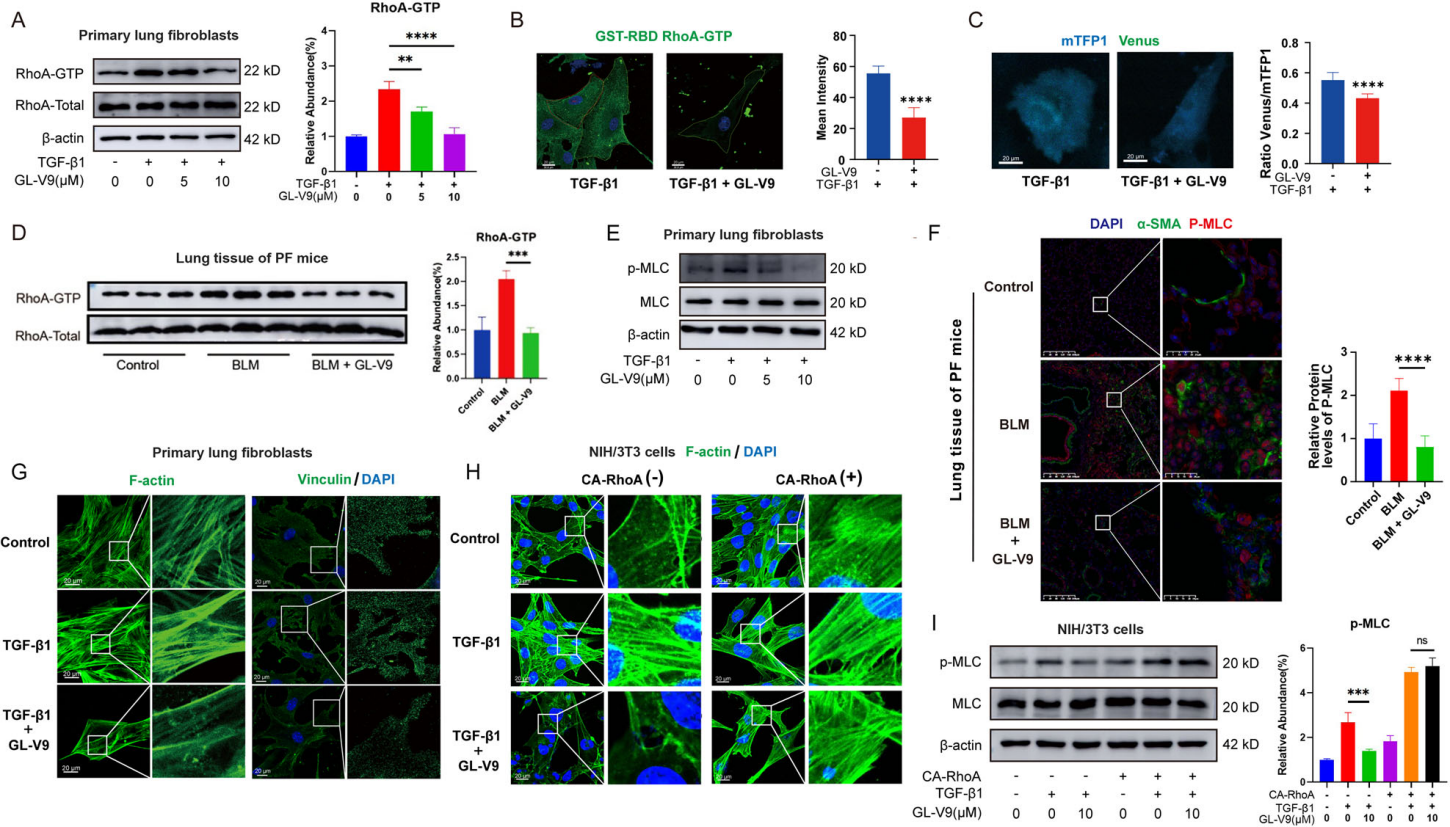
GL-V9 inhibits RhoA activation in myofibroblasts and impedes the reorganization of the actin cytoskeleton. **A**–**C**, **G** Following a 24-h serum starvation period, the primary lung fibroblasts from mice were exposed to 5 ng/mL TGF-β1 for 24 h while concurrently administering GL-V9 treatment. **A** The GTP-bound form of RhoA captured by GST-RBD was assayed by Western blotting and quantified. **B** Immunofluorescence assays employed GST-RBD to specifically target and visualize the GTP-bound RhoA within the cells. Scale bar: 20 μm. The mean intensity of immunofluorescence was quantified. Differences between groups were analyzed using unpaired t-test. **C** RhoA activity detection using the biosensor RhoA2G through Förster resonance energy transfer (FRET). Scale bar: 20 μm. Venus/mTFP1 ratio images with fluorescence reflecting RhoA activity was quantified. Differences between groups were analyzed using unpaired t-test. **D** The active RhoA protein captured with the histone lysate of fresh lung tissues of bleomycin-induced pulmonary fibrosis (PF) mice. **E** The primary lung fibroblasts were subjected to serum starvation for 24 h, followed by treatment with GL-V9 for 24 h and a pre-collection treatment of 10 ng/mL TGF-β1 for 15 min before cell harvesting. The phosphorylation levels of MLC. **F** Dual immunohistochemistry of α-SMA (marker of myofibroblasts) and P-MLC in lung tissue of mice with bleomycin-induced PF, with quantification of P-MLC fluorescence intensity. Scale bar for the main image: 200 μm; enlarged section scale bars: 25 μm. **G** The F-actin cytoskeleton and the focal adhesions in primary lung fibroblasts from mice, with F-actin labeled by phalloidin and focal adhesions marked by vinculin antibodies. Scale bar: 20 μm. **H** The effect of GL-V9 and constitutively active (CA) RhoA expression on the F-actin cytoskeleton in NIH/3T3. NIH/3T3 cells were treated with 10 μM GL-V9 in serum-free culture medium for 24 h, followed by 10 ng/mL TGF-β1 for 1 h. Scale bar: 20 μm. **I** The effect of CA RhoA expression on the phosphorylation levels of MLC in GL-V9-treated NIH/3T3. NIH/3T3 cells were treated with 10 μM GL-V9 in serum-free culture medium for 24 h, followed by 10 ng/mL TGF-β1 for 15 min. Data are shown as mean ± SEM. ns *p* > 0.05, **p < 0.01, ***p < 0.001, ****p < 0.0001.

We hypothesized that the action of GL-V9 in hindering the binding of RhoGEFs to RhoA could inhibit the activation of RhoA in myofibroblasts. To investigate this, we employed the GST-tagged Rho binding domain (RBD) to isolate the active GTP-bound form of RhoA from cellular extracts or to visualize the distribution of RhoA-GTP within cells. TGF-β1 is the primary cytokine that promotes the transformation of normal lung fibroblasts into myofibroblasts. GL-V9 treatment resulted in a decreased level of RhoA-GTP in TGF-β1-induced myofibroblasts **(**Fig. 4A, B). RhoA2G is a biosensor designed to detect intracellular RhoA activation based on Förster resonance energy transfer (FRET) technology [38]. GL-V9 significantly reduced the ratio of Venus fluorescence to mTFP1 fluorescence and the incidence of FRET, thereby decreasing RhoA activity (Fig. 4C). Moreover, the active RhoA protein was captured with the histone lysate of fresh lung tissues of bleomycin-induced PF mice. The results demonstrated that the activity of lung histone RhoA was significantly enhanced in the BLM group of a bleomycin-induced lung fibrosis model, and the intervention of GL-V9 was found to effectively inhibit the enhancement of RhoA activity (Fig. 4D). The activation of ROCKs is dependent on the activity of RhoA, which functions by phosphorylating several cytoskeletal regulatory proteins, including MLC, LIMK, and Ezrin [39]. GL-V9 treatment resulted in a significant reduction in the phosphorylation levels of MLC in myofibroblasts cultured in vitro as well as within the lung tissue of mice with bleomycin-induced PF, thereby decreasing the activity of ROCKs (Fig. 4E, F). Activation of RhoA-ROCKs can induce the formation of actin stress fibers and focal adhesions, facilitating the reorganization of the cytoskeleton. GL-V9 led to the disassembly of F-actin, reducing actin stress fibers and resulting in a disorganized cytoskeletal microfilament network (Fig. 4G, H). Furthermore, GL-V9 inhibited the formation of focal adhesions in myofibroblasts, which is marked by the core focal adhesion proteins vinculin (Fig. 4G). To determine whether the impact of GL-V9 on the morphology of the actin cytoskeleton is mediated by its inhibition of RhoA activity, we engineered NIH/3T3 cells to express constitutively active RhoA (CA RhoA). The expression of CA RhoA assists cells in maintaining the integrity of the actin cytoskeleton under serum-free conditions. GL-V9 was unable to promote the disassembly of the actin cytoskeleton in NIH/3T3 cells expressing CA RhoA (Fig. 4H). Similarly, GL-V9 is unable to reduce the phosphorylation levels of MLC in cells expressing CA RhoA (Fig. 4I). These results indicate that GL-V9 inhibits RhoA activation and impedes the reorganization of the actin cytoskeleton in myofibroblasts via the RhoA-ROCK pathway.

### GL-V9 suppresses myofibroblasts activation through the reorganization of cytoskeleton and the inactivation of MRTF and YAP

**Fig. 5:**
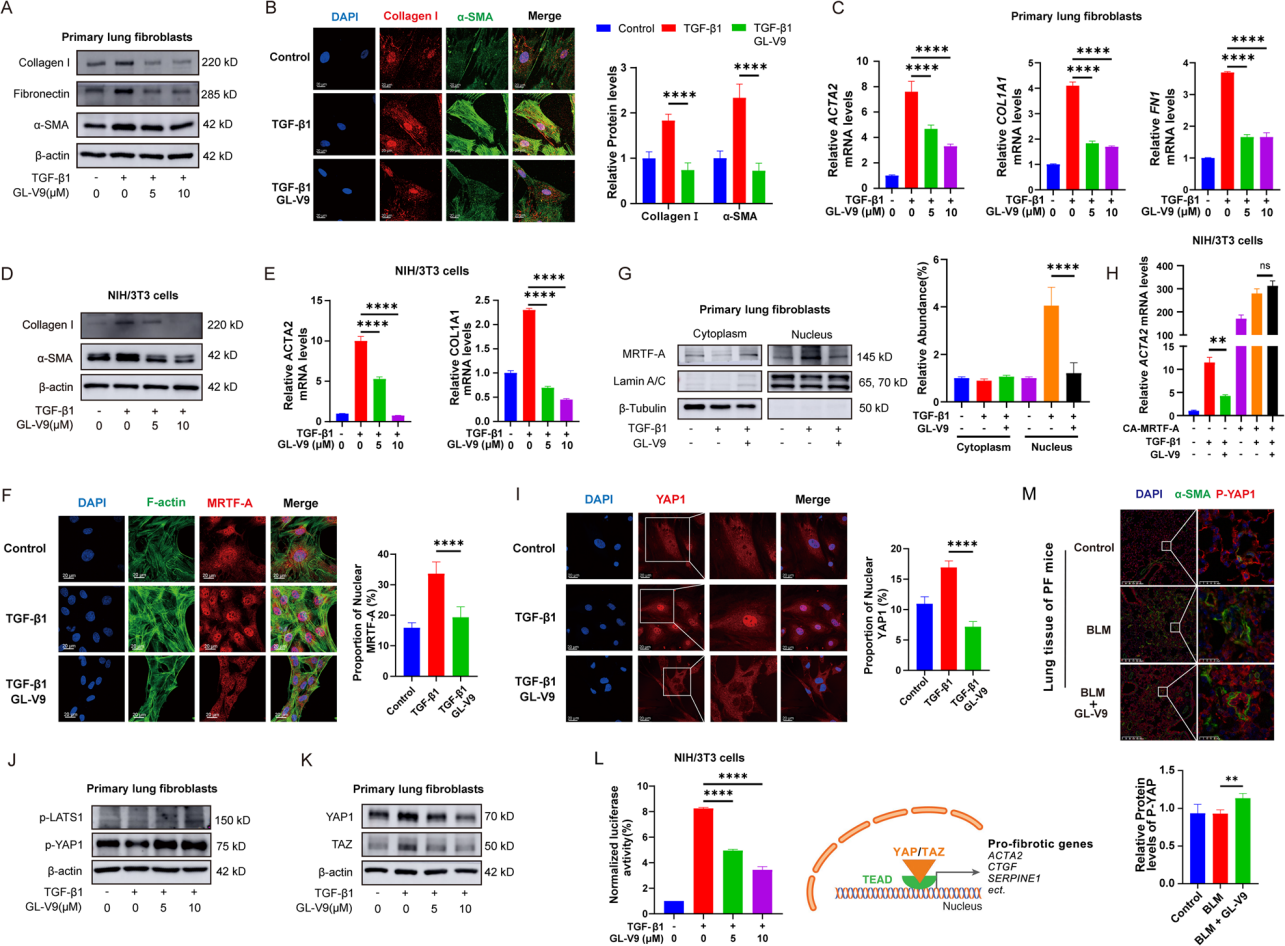
GL-V9 inhibits the reorganization of the actin cytoskeleton and affects the activity of MRTF and YAP. The primary lung fibroblasts from mice were subjected to serum starvation for 24 h, followed by treatment with 5 ng/mL TGF-β1 and GL-V9 for various times. NIH/3T3 cells were treated with 10 ng/mL of TGF-β1 in a serum-free culture medium for 24 h and treated with GL-V9. **A** The protein expression of α-SMA, collagen I, and fibronectin in primary lung fibroblasts (48 h). **B** The content and distribution of Collagen I and α-SMA in primary lung fibroblasts (48 h) were examined via immunofluorescence. Scale bar: 20 μm. **C** The mRNA content of *ACTA2*, *COL1A1*, and *FN1* in primary lung fibroblasts (24 h). **D** The protein expression of α-SMA and collagen I in NIH/3T3 cells. **E** The mRNA content of *ACTA2* and *COL1A1* in NIH/3T3 cells. **F** The nuclear localization of MRTF-A and the F-actin cytoskeleton in primary lung fibroblasts (24 h). Scale bar: 20 μm. The proportion of nuclear MRTF-A was quantified. **G** The distribution of MRTF-A within the primary lung fibroblasts was assessed through nucleocytoplasmic fractionation experiments (24 h). **H** The effect of CA MRTF-A expression on the content of *ACTA2* mRNA in NIH/3T3 cells. Differences between groups were analyzed using Welch’s ANOVA. **I** The nuclear localization of YAP in primary lung fibroblasts (12 h). Scale bar: 20 μm. The proportion of nuclear YAP1 was quantified. **J** The phosphorylation levels of YAP1 and LATS in primary lung fibroblasts (12 h). **K** The protein level of YAP1 and LATS in primary lung fibroblasts (24 h). **L** Dual-luciferase assay to measure TEAD transcriptional activity. Expression of a YAP/TAZ-responsive synthetic promoter driving firefly luciferase, along with a control Renilla luciferase, in NIH3T3 cells. **M** Dual immunohistochemistry of α-SMA and P-YAP1 in lung tissue of mice with bleomycin-induced pulmonary fibrosis, with quantification of P-YAP1 fluorescence intensity. Scale bar for the main image: 200 μm; enlarged section scale bars: 25 μm. Data are shown as mean ± SEM. ns *p* > 0.05, **p < 0.01, ***p < 0.001, ****p < 0.0001.

Mature myofibroblasts are characterized by high expression of α-SMA, robust synthesis, and secretion of substantial amounts of extracellular matrix proteins, and possession of a well-developed cytoskeleton and focal adhesions. GL-V9 treatment suppressed the protein expression as well as mRNA expression of α-SMA (*ACTA2* gene), collagen I (*COL1A1* gene), and fibronectin in TGF-β1-induced myofibroblasts (Fig. 5A–E). Given the previously demonstrated ability of GL-V9 to impede cytoskeletal remodeling, GL-V9 treatment removes key features of myofibroblast maturation, indicating its potential to inhibit myofibroblast activation.

The actin cytoskeleton is regarded as a critical component in pro-fibrotic signaling pathways, influencing the subcellular localization and activity of the transcriptional coactivators myocardin-related transcription factor (MRTF) and yes-associated protein (YAP), thereby regulating the expression of genes associated with fibrosis [40, 41]. The polymerization of F-actin reduces the availability of G-actin, which normally confines MRTF to the cytoplasm. This enables MRTF to translocate to the nucleus, where it cooperates with Serum Response Factor (SRF) to enhance the transcription of pro-fibrotic genes such as α-SMA and connective tissue growth factor (CTGF). GL-V9 induced the depolymerization of F-actin and a marked reduction in the nuclear accumulation of MRTF-A (Fig. 5F, G). The elimination of the RPEL motifs that bind to G-actin and the introduction of an additional nuclear localization sequence (NLS) render MRTF-A constitutively active. In NIH/3T3 cells expressing MRTF-A, GL-V9 failed to decrease *ACTA2* mRNA levels. This indicates that the inhibitory effect of GL-V9 on *ACTA2* expression is dependent on its impact on the subcellular localization of MRTF-A (Fig. 5H). Hippo signaling pathway is essential for detecting alterations in the actin cytoskeleton and mediating intracellular mechanical signal transduction. Inhibition of the Hippo pathway reduces the phosphorylation levels of Lats1/2, which are upstream kinases in the YAP/TAZ signaling cascade. Consequently, YAP/TAZ translocate to the nucleus in their non-phosphorylated form and interact with the TEAD1-4 transcription factors to promote the transcription of genes that drive fibrosis [42, 43]. GL-V9 hindered the nuclear localization of YAP1 and increased the phosphorylation of both YAP and LATS, indicating the inhibition of YAP activity and the activation of the Hippo pathway (Fig. 5I, J). Subsequently, phosphorylated YAP and TAZ are then marked for ubiquitination and degradation by the proteasome. Thus, GL-V9 significantly reduced the protein levels of YAP/TAZ (Fig. 5K). Correspondingly, GL-V9 increased the phosphorylation of YAP in myofibroblasts within the lungs of mice with bleomycin-induced PF (Fig. 5M). Furthermore, dual-luciferase reporter assays demonstrated that GL-V9 substantially diminishes the activation of the transcription factor TEAD by YAP (Fig. 5L). These results indicate that GL-V9 inhibits the activation of myofibroblasts by suppressing the RhoA-ROCK pathway, thereby impeding the reorganization of the actin cytoskeleton and inhibiting the activity of MRTF and YAP.

**Fig. 6:**
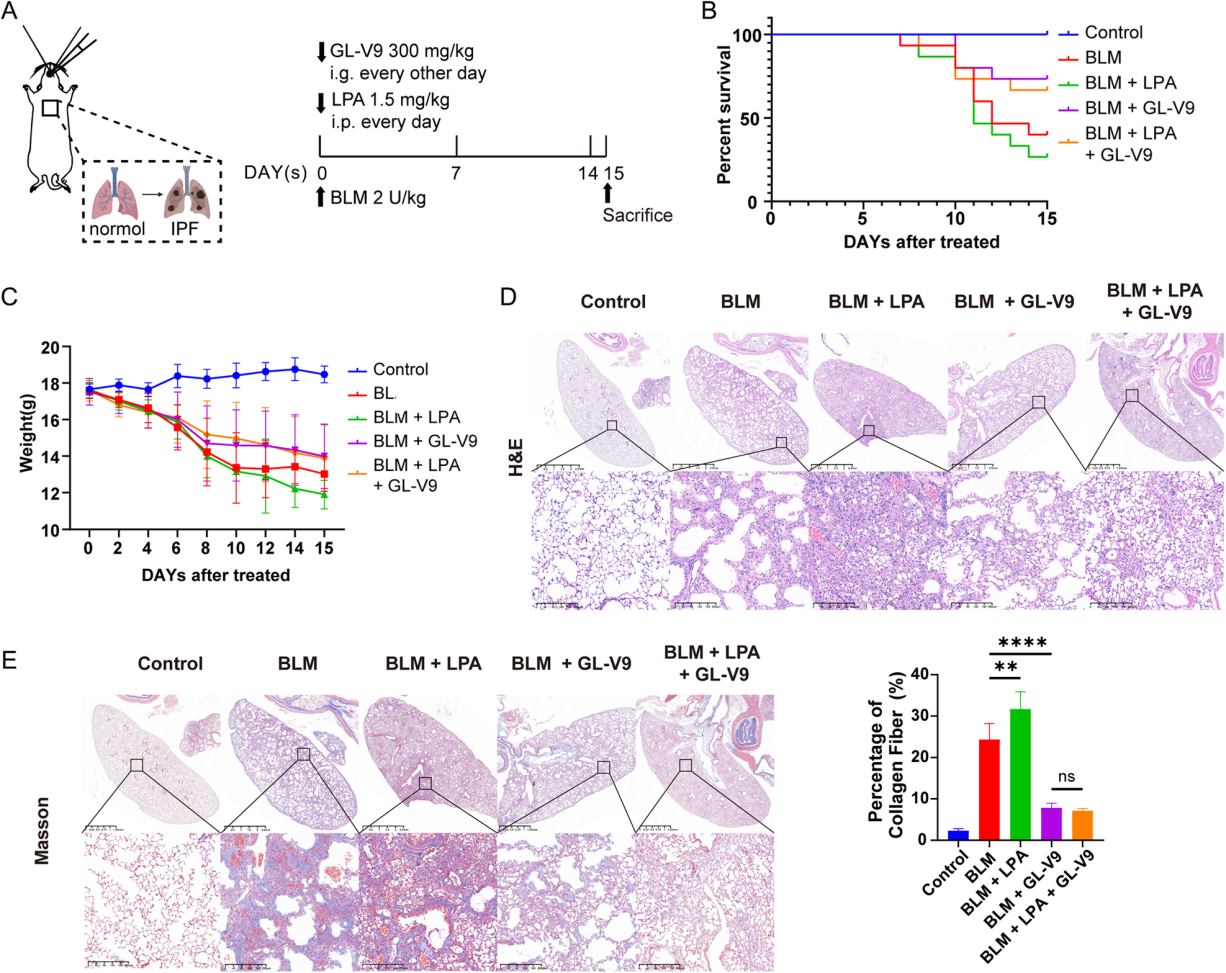
GL-V9 antagonizes the pro-fibrotic effects of Rho GTPases agonist lysophosphatidic acid (LPA) in mice with bleomycin-induced pulmonary fibrosis. **A** Schematic representation of the experimental design for evaluating the effects of GL-V9 and LPA on bleomycin-induced pulmonary fibrosis in mice. Pulmonary fibrosis was induced by transoral instillation of bleomycin at a dose of 2 U/kg. GL-V9 was administered at a dose of 300 mg/kg every other day, starting on the day bleomycin administration. LPA was administered at a dose of 1.5 mg/kg daily, starting on the day bleomycin administration. The study was conducted in five groups: the saline control group (Control, *n* = 7), the bleomycin-induced pulmonary fibrosis model group (BLM, *n* = 15), the group treated with LPA (BLM + LPA, *n* = 15), the group treated with GL-V9 (BLM + GL-V9, *n* = 15), and the group treated with LPA and GL-V9 (BLM + LPA + GL-V9, *n* = 15). **B** Survival curve of mice. **C** Weight changes in mice. **D**, **E** H&E stains and Masson stains of mouse lung tissues. In Masson staining, collagen fibers appear blue-green, while the cytoplasm is stained red. The quantification of collagen fiber area in Masson-stained sections is shown. Scale bar for the main image: 2.5 mm; enlarged section scale bars: 100 µm. Data are shown as mean ± SEM. ns *p* > 0.05, **p < 0.01, ***p < 0.001, ****p < 0.0001.

Therefore, the small-molecule inhibitor GL-V9, which targets Rho GEFs, effectively inactivates RhoA and inhibits the RhoA-ROCK signaling pathway. This mechanism subsequently prevents the activation of myofibroblasts and the progression of PF. To confirm the underlying mechanism of GL-V9 against PF in vivo, we used lysophosphatidic acid (LPA) acts as an endogenous agonist for RhoA (Fig. 6A), which plays a crucial role in fibrosis by promoting fibroblast activation and extracellular matrix remodeling [44]. LPA intensified bleomycin-induced pulmonary fibrosis in mice models; however, GL-V9 effectively antagonized the pro-fibrotic effects of LPA and still demonstrated significant therapeutic efficacy in the presence of both LPA and bleomycin. Both the survival rates and weight loss trends of PF mice were significantly improved (Fig. 6B, C). More severe pathological fibrosis and collagen deposition in mouse lungs caused by co-administration of LPA and bleomycin were also reversed by GL-V9 (Fig. 6D, E). Thus, GL-V9 exerts its anti-pulmonary fibrosis effects by inhibiting RhoA activity.

### GL-V9 suppresses M2 polarization of profibrotic macrophages by inactivating RhoA-STAT3 signaling pathway

**Fig. 7:**
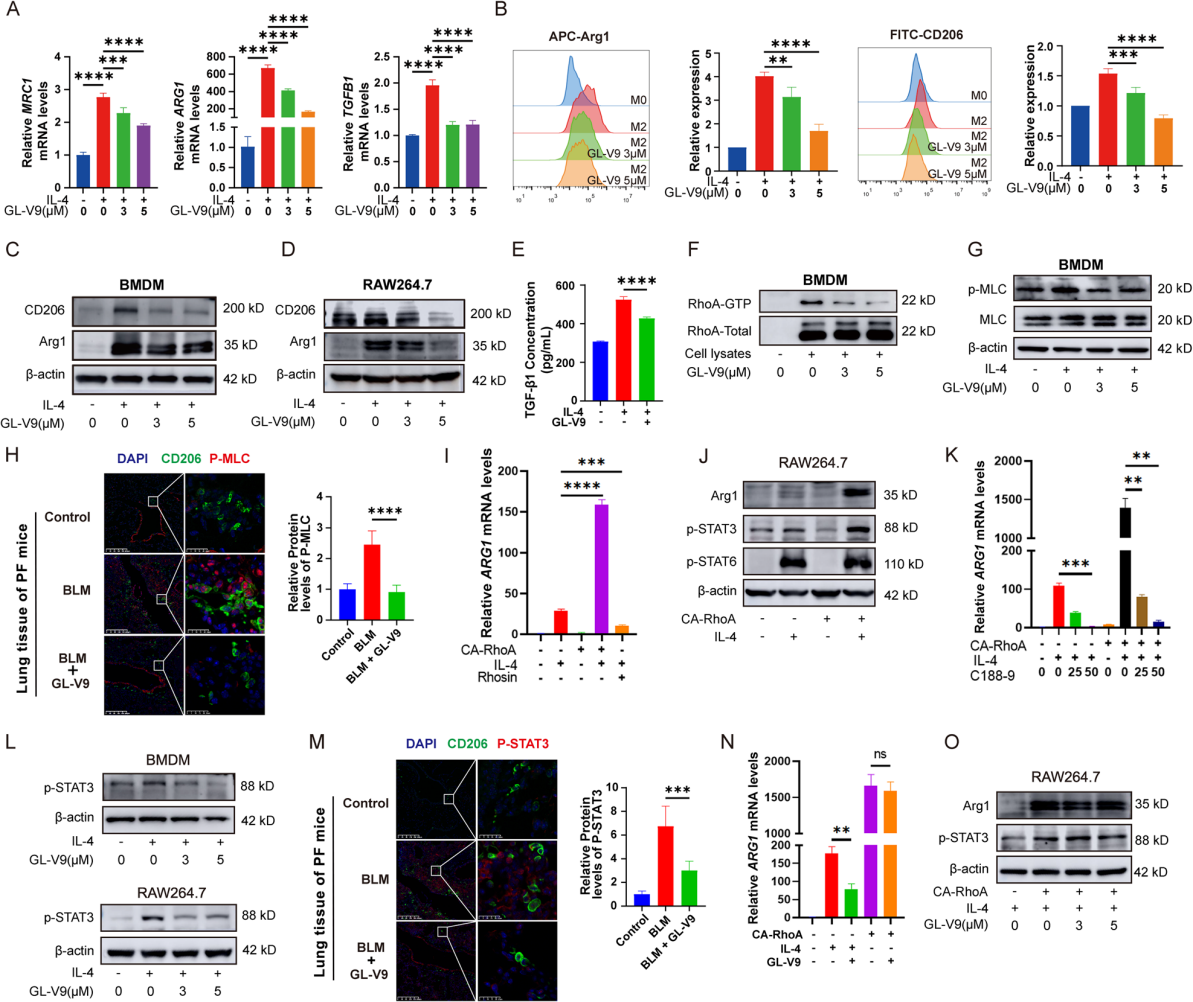
GL-V9 inhibits M2 polarization of macrophages by suppressing the RhoA-STAT3 signaling pathway. **A**–**C**, **F**, **G** Bone marrow-derived macrophages (BMDMs) were treated with 20 ng/mL IL-4 and GL-V9 for 48 h. **A** The mRNA levels of M2 polarization phenotype. **B** The flow cytometry results showed the expression levels of M2 polarization markers Arg1 and CD206. **C** The protein expressions of the M2 polarization phenotype. **D** RAW264.7 cells were treated with 20 ng/mL IL-4 and GL-V9 for 48 h. The protein expression of the M2 polarization phenotype. **E** The concentration of TGF-β1 in the cell culture supernatant. BMDMs were treated with 20 ng/mL IL-4 and GL-V9 for 48 h, followed by incubation in a serum-free medium for 48 h. **F** The GTP-bound form of RhoA captured by GST-RBD. **G** The phosphorylation levels of MLC. **H** Dual immunohistochemistry of CD206 and P-MLC in lung tissue of mice with bleomycin-induced pulmonary fibrosis, with quantification of P-MLC fluorescence intensity. Scale bar for the main image: 200 μm; enlarged section scale bars: 25 μm. **I** The effect of CA RhoA expression and Rhosin on the mRNA levels of *ARG1* in RAW264.7. RAW264.7 were treated with 20 ng/mL IL-4 and Rhosin for 48 h. Differences between groups were analyzed using Welch’s ANOVA. **J** The effect of CA RhoA expression on the protein expression of Arg1 and the levels of p-STAT3 and p-STAT6. RAW264.7 were treated with 20 ng/mL IL-4 for 48 h. **K** The effect of CA RhoA expression and the STAT3 inhibitor C188-9 on the mRNA levels of *ARG1* in RAW264.7 cells. Differences between groups were analyzed using Welch’s ANOVA. **L** The phosphorylation of STAT3. BMDMs and RAW264.7 were treated with 20 ng/mL IL-4 and GL-V9 for 48 h. **M** Dual immunohistochemistry of CD206 and P-STAT3 in lung tissue of mice with bleomycin-induced pulmonary fibrosis, with quantification of P-STAT3 fluorescence intensity. Scale bar for the main image: 200 μm; enlarged section scale bars: 25 μm. **N** The effect of GL-V9 and CA RhoA expression on the mRNA levels of *ARG1* in RAW264.7. RAW264.7 were treated with 20 ng/mL IL-4 and 5 μM GL-V9 for 48 h. Differences between groups were analyzed using Welch’s ANOVA. **O** The effect of GL-V9 and CA RhoA expression on the protein expression of Arg1 and the phosphorylation of STAT3 and STAT6. RAW264.7 were treated with 20 ng/mL IL-4 and GL-V9 for 48 h. Data are shown as mean ± SEM. ns *p* > 0.05, **p < 0.01, ***p < 0.001, ****p < 0.0001.

Macrophage polarization is strongly correlated with pulmonary fibrosis, and M2 polarization significantly promotes the fibrotic process. In the IL-4-induced mouse bone marrow-derived macrophage (BMDM) M2 polarization model, GL-V9 significantly reduced the protein and mRNA expression levels of M2 polarization markers, including CD206 (*MRC1 g*ene), Arg1 (*ARG1 g*ene), and TGF-β1 (*TGFB1 g*ene). (Fig. 7A–C). Similarly, GL-V9 reduced the expression of M2 polarization molecular markers in the IL-4-induced RAW264.7 cell model (Fig. 7D, N). TGF-β1 is a key pro-fibrotic molecule secreted by M2-type macrophages [45], and the treatment with GL-V9 inhibited the secretion of TGF-β1 by BMDM (Fig. 7E). These findings suggest that GL-V9 suppresses M2 polarization of profibrotic macrophages in vitro models.

In further studies, GL-V9 was observed to reduce the levels of active RhoA-GTP and phosphorylated MLC in BMDM (Fig. 7F, G), indicating that GL-V9 decreases RhoA-ROCK activity in macrophages. Similarly, GL-V9 reduced the phosphorylation of MLC in M2 macrophages with the lung tissues of mice with bleomycin-induced PF (Fig. 7H). To assess whether the dual effects of GL-V9—namely, the inhibition of M2 polarization and the suppression of RhoA-ROCK activity in macrophages—are interconnected, we introduced constitutively active RhoA into RAW264.7 cells. Our preliminary results indicated that under IL-4 stimulation, the presence of constitutively active RhoA increased the mRNA expression of *Arg1*, thereby promoting cellular polarization (Fig. 7I). Conversely, treatment with the RhoA activity inhibitor Rhosin resulted in a reduction in macrophage polarization (Fig. 7I), indicating a correlation between alterations in RhoA activity and M2 polarization in macrophages. The JAK/STAT signaling pathway is known to regulate macrophage polarization. Activation of STAT3/STAT6 promotes macrophage M2 polarization [46, 47]. In the presence of IL-4, RAW264.7 cells expressing CA RhoA exhibited a significant increase in p-STAT3 levels compared to wild-type cells, which is consistent with the expression trends of Arg1 (Fig. 7J). The use of STAT3 inhibitor C188-9 effectively blocked the enhancement of *Arg1* mRNA expression by CA RhoA (Fig. 7K). This suggests that RhoA signaling may regulate macrophage polarization through STAT3. GL-V9 reduced p-STAT3 levels in mouse bone marrow-derived macrophages, and RAW264.7 cells, as well as in M2 macrophages with the lung tissues of mice with bleomycin-induced PF (Fig. 7L, M). GL-V9 was unable to reduce *Arg1* mRNA expression and decreased STAT3 phosphorylation levels in RAW264.7 cells expressing CA RhoA (Fig. 7N, O). These findings suggest that the inhibitory effect of GL-V9 on the M2 polarization of macrophages is dependent on the RhoA-STAT3 signaling axis.

## Discussion

IPF typically presents with progressively worsening dyspnea and significantly reduced lung compliance [48], often leading to a prognosis worse than many malignancies. Treatment guidelines recommend Nintedanib and Pirfenidone to slow disease progression and lung function decline, but these drugs have limited effects on improving patient quality of life and face resistance issues [49, 50]. Lung transplantation is considered the only curative option, but its applicability is limited by factors such as patient age, comorbidities, donor shortages, and potential rejection, making it accessible to only a few patients [51, 52]. The development of new drugs is challenging due to incomplete understanding of IPF pathogenesis, differences between animal models and human IPF, and significant variability in disease progression among patients [53]. In this study, we extend the therapeutic application of a novel GEFs inhibitor GL-V9 to pulmonary fibrosis and investigate its underlying mechanisms.

Targeting the Rho GTPase family and its downstream effector ROCK has been proven to be a reliable therapeutic strategy in various fibrotic diseases [54, 55]. Multiple studies have demonstrated the efficacy of the ROCK inhibitor Fasudil and Y-27632 in treating pulmonary fibrosis [56, 57]. However, the globular structure of the Rho GTPase family lacks appropriate binding sites for drugs, resulting in a scarcity of research on the application of corresponding drugs for fibrosis treatment. Rho GEFs, key activators of Rho GTPases, through their specific interactions with Rho GTPase family, present more suitable targets for pharmacological intervention than Rho GTPase itself. The Dbl family of Rho GEFs typically incorporates the DH-PH domain; the DH domain drives the guanine nucleotide exchange and imparts substrate specificity, whereas the PH domain anchors these GEFs to the membrane and assists in their association with the respective GTPases [58]. This study confirmed that GL-V9 interacts with the DH/PH domain of LARG, forming hydrogen bonds with key residues Arg922, Leu924, and Lys939, which are critical for RhoA binding and activation.

The Rho GTPase family plays a critical role in cytoskeletal reorganization and cell motility [59], which are closely associated with myofibroblast activation. Myofibroblasts, key effector cells in fibrosis, synthesize and secrete excess ECM proteins, possess a developed cytoskeleton and mature focal adhesions, and exert mechanical forces on the ECM, leading to irreversible contraction and collagen fiber reorganization. This process disrupts normal alveolar structure and impairs organ function. The reorganization of the actin cytoskeleton is crucial for regulating the subcellular distribution of transcriptional coactivators MRTF and YAP, promoting the transcription of fibrotic genes such as α-SMA, CTGF, and PAI-1 (plasminogen activator inhibitor-1). Targeting the Rho GTPase family is thus a promising strategy to inhibit myofibroblast activation. This study found that GL-V9 treatment impedes key features of myofibroblast maturation and reduces the levels of myofibroblast marker proteins through inhibiting Rho GEFs-RhoA pathway. GL-V9 induces actin cytoskeleton disassembly and reduces focal adhesion complexes, inhibiting the activity of MRTF and YAP, which leads to decreased transcription of pro-fibrotic genes and effectively inhibits myofibroblast activation and associated fibrotic effects.

Initiating pharmacological interventions following the resolution of the acute inflammatory response can yield a more precise evaluation of a drug’s antifibrotic potential. GL-V9 effectively arrests fibrotic progression, whether during the inflammation phases or fibroproliferation and tissue remodeling phases. Its therapeutic mechanisms in animal models include inhibiting inflammatory responses, preventing myofibroblast differentiation, and reducing macrophage M2 polarization. Although immunosuppressants have had limited success in IPF clinical trials, the inflammatory process is crucial in both physiological wound healing and pathological fibrosis [60]. In IPF, a pro-fibrotic inflammatory environment, dominated by abundant M2 macrophages and Th2 lymphocytes, characterizes the lungs. M2 macrophages secrete pro-fibrotic cytokines, playing a key role in promoting pulmonary fibrosis [61]. This study confirmed that GL-V9 effectively reduces M2 macrophage polarization in both in vivo and in vitro models by inhibiting the Rho GEFs-RhoA pathway. Previous research suggested that ROCK signaling inhibition affects M2 polarization by blocking STAT3 activity. This study further confirms that inhibiting the Rho GEFs-RhoA pathway effectively blocks STAT3 activation, thereby interfering with M2 macrophage polarization. However, more detailed signaling mechanisms require further investigation.

**Fig. 8:**
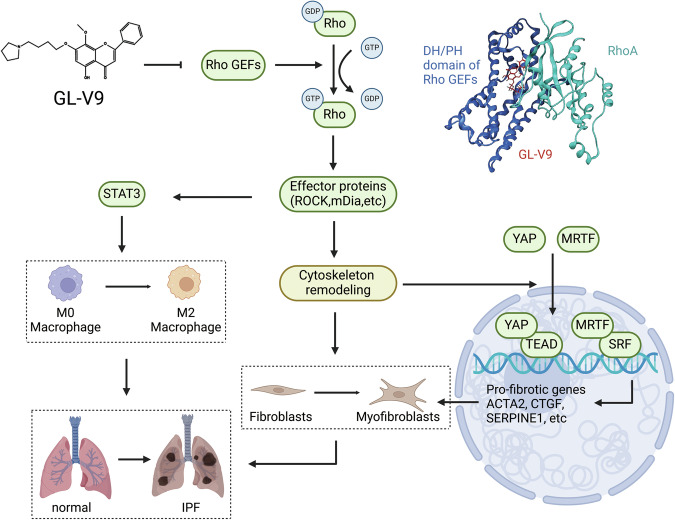
A proposed schematic diagram for Rho GEFs inhibitor GL-V9 in alleviation of pulmonary fibrosis through the dual modulations of myofibroblast activation and profibrotic macrophage polarization. By binding to the DH/PH domain of Rho GEFs, GL-V9 suppresses Rho GTPase-dependent myofibroblast activation by interfering with cytoskeletal reorganization and inactivating MRTF and YAP, and inhibits M2 polarization of profibrotic macrophage by inactivating STAT3.

This study demonstrates that Rho GEFs inhibitor GL-V9 significantly attenuates the progression of pulmonary fibrosis in a bleomycin-induced mouse model. GL-V9 acts by binding to the DH/PH domain of Rho GEFs, thereby impeding their ability to activate RhoA. This interaction effectively inhibits the RhoA-ROCK signaling pathway, which is critical in preventing the activation of myofibroblasts and the M2 polarization of macrophages, leading to the mitigation of pulmonary fibrosis (Fig. 8). The findings position GL-V9 as a viable therapeutic candidate and validate the approach of targeting the Rho GEFs signaling in the treatment of pulmonary fibrosis.

## Supplementary information

Supplementary Materials and Methods

Supplementary Figures

Supplementary Table

Original Data

## Data Availability

The data that support the findings of this study are present in the paper or the Supporting Information. Any additional information required for reanalysis is available from the corresponding authors upon reasonable request.
