# Supplementary material for: Inhibition of Rho GEFs attenuates pulmonary fibrosis through suppressing myofibroblast activation and reprogramming profibrotic macrophages
Source: Cell Death Dis. 2025 Apr 11;16(1):278. doi: 10.1038/s41419-025-07573-5 (PMC11992128; doi:10.1038/s41419-025-07573-5)
Supplement: Supplementary file 1 — Supplementary Materials and Methods [file 41419_2025_7573_MOESM1_ESM.pdf]

Supplementary Materials and Methods

Western blot

Cells and tissues were lysed in RIPA Lysis and Extraction Buffer (Thermo Fisher Scientific, USA) with protease and phosphatase inhibitors (GlpBio, USA), and protein concentrations were determined using the Pierce BCA Protein Assay Kit (Thermo Fisher Scientific, USA). Samples containing equal amounts of total protein were separated on SDS-PAGE gels of appropriate concentration and transferred to NC membranes (Millipore, USA). Membranes were incubated with primary antibodies at 4°C overnight, with β-actin serving as a loading control. HRP-conjugated secondary antibodies were incubated with the membranes at room temperature. ECL substrate was used for detection, and bands were imaged using the Amersham Imager 600 chemiluminescence imaging system (GE, USA). Quantification was performed using ImageJ software.

Quantitative real-time PCR

Total RNA from cells and tissues was extracted using FreeZol Reagent (Vazyme, China) and reverse transcribed into cDNA using the HiScript III 1st Strand cDNA Synthesis Kit (Vazyme, China). Quantitative real-time PCR (qRT-PCR) analysis was conducted on a QuantStudio 3 Real-Time PCR System (Thermo Fisher Scientific, USA) with AceQ qPCR SYBR Green Master Mix (Vazyme, China). Relative expression levels were calculated and normalized to the GAPDH gene using the 2–ΔΔCt method. Oligonucleotides used in this study for PCR are listed in Information supplementary material Table S1.

Flow Cytometry Analysis

To collect bronchoalveolar lavage fluid (BALF) from mice, the animals were anesthetized, and the trachea was exposed. A pipette tip was inserted into the trachea, and the lungs were lavaged with pre-cooled PBS buffer. The lavage fluid was centrifuged to obtain cells for flow cytometry analysis. After drug treatment, bone marrow-derived macrophages (BMDMs) were digested with trypsin and centrifuged to collect cells for flow cytometry. Each sample was incubated with 1 μg of anti-mouse CD16/32 (Elabscience, China) to block Fc receptors. The cells were then fixed with a cell fixation buffer for 30 minutes, followed by permeabilization and incubation with flow cytometry antibodies

for 30 minutes. APC-F4/80 and PE-CD206 antibodies were purchased from eBioscience (USA), and FITC-CD206 was obtained from Elabscience (China). The samples were analyzed using an Accuri C6 Plus flow cytometer (BD, USA), and the results were processed using FlowJo software.

Cellular Thermal Shift Assay (CETSA)

NIH/3T3 cells were transfected with lentivirus encoding GST-ARHGEF12(765-1138aa) or GST-ARHGEF12(765-1138aa)-922A, followed by selection with puromycin to establish stable cell lines. Cells were treated with 75 μM GL-V9 for 3 hours and then collected. The cell pellets were resuspended in PBS containing protease inhibitors and distributed equally into 200 μL tubes. These samples were heated at different temperatures using a PCR machine: 37°C, 41.7°C, 45.4°C, 47.3°C, 50.7°C, and 52°C for 3 minutes each. After heating, the cells were lysed by repeated freeze-thaw cycles using liquid nitrogen, and the supernatant was collected by centrifugation. Protein thermal stability was assessed by Western blotting using an anti-GST antibody.

Nuclear and Cytoplasmic Fractionation

Nuclear and cytoplasmic fractionation was performed using a kit from Beyotime (Shanghai, China). Cells were collected and resuspended in a cytoplasmic protein extraction reagent containing phenylmethylsulfonyl fluoride (PMSF). After centrifugation, the supernatant, which contained the cytoplasmic proteins, was collected. The pellet was resuspended in a nuclear protein extraction reagent, which was supplemented with PMSF. The nuclei were lysed using an ultrasonic homogenizer set at 100 W power, with a cycle of 3 seconds on and 5 seconds off, for a total duration of 3 minutes. After centrifugation, the supernatant containing the nuclear proteins was collected. Subsequent detection was performed by Western blotting, using LaminA/C as the nuclear marker and β-Tubulin as the cytoplasmic marker.
