# Supplementary material for: Inhibition of Rho GEFs attenuates pulmonary fibrosis through suppressing myofibroblast activation and reprogramming profibrotic macrophages
Source: Cell Death Dis. 2025 Apr 11;16(1):278. doi: 10.1038/s41419-025-07573-5 (PMC11992128; doi:10.1038/s41419-025-07573-5)
Supplement: Supplementary file 2 — Supplementary Figures [file 41419_2025_7573_MOESM2_ESM.pdf]

Supplementary Figures

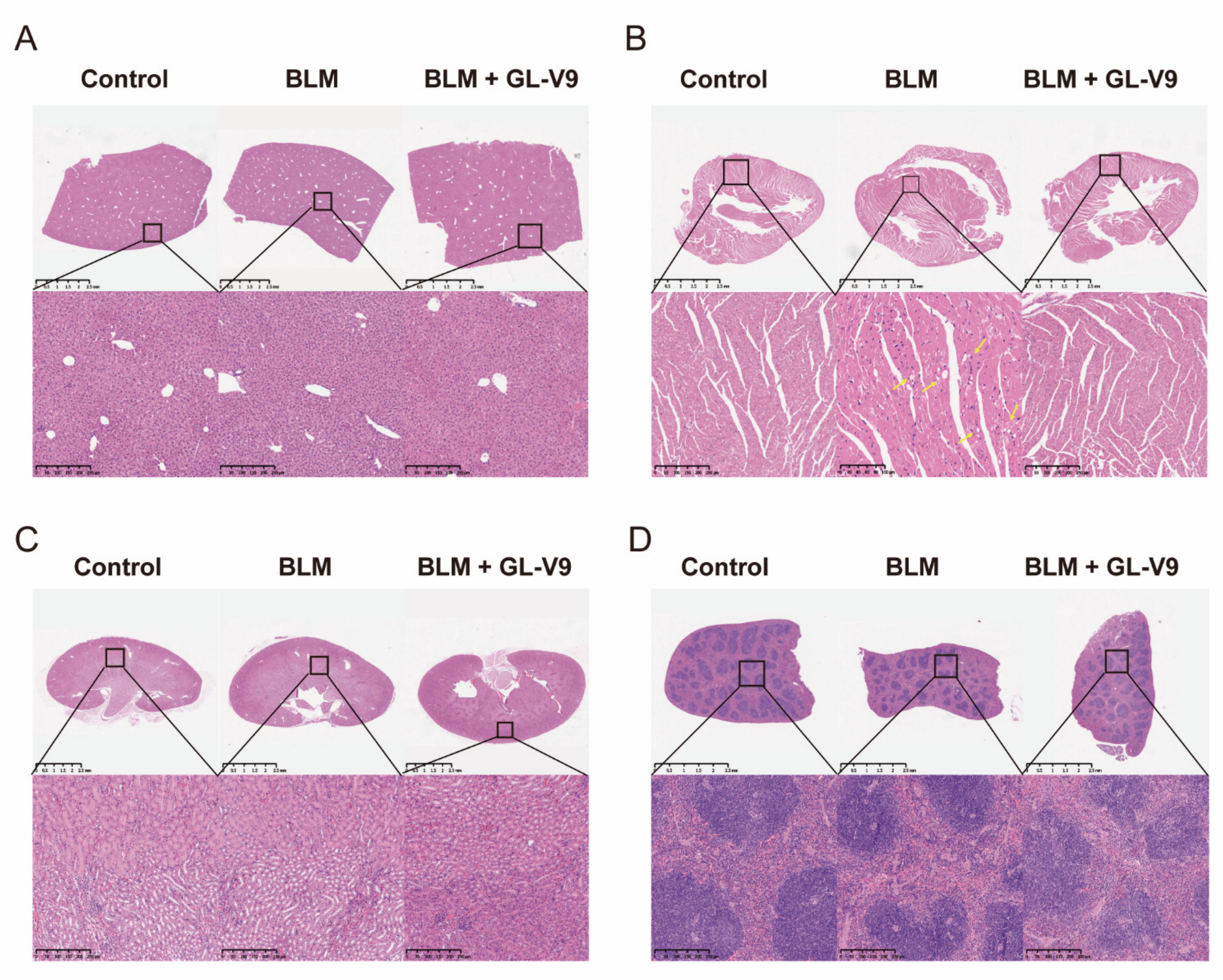

Figure S1 Pathological analysis of the liver (A), heart (B), kidney (C), and spleen (D) from mice with pulmonary fibrosis induced by transoral instillation of bleomycin and treated with GL-V9 on the 21st day following model establishment. Yellow arrows indicate mild lesions. Scale bar for the main image: 2.5 mm; enlarged section scale bars: 250 μm.

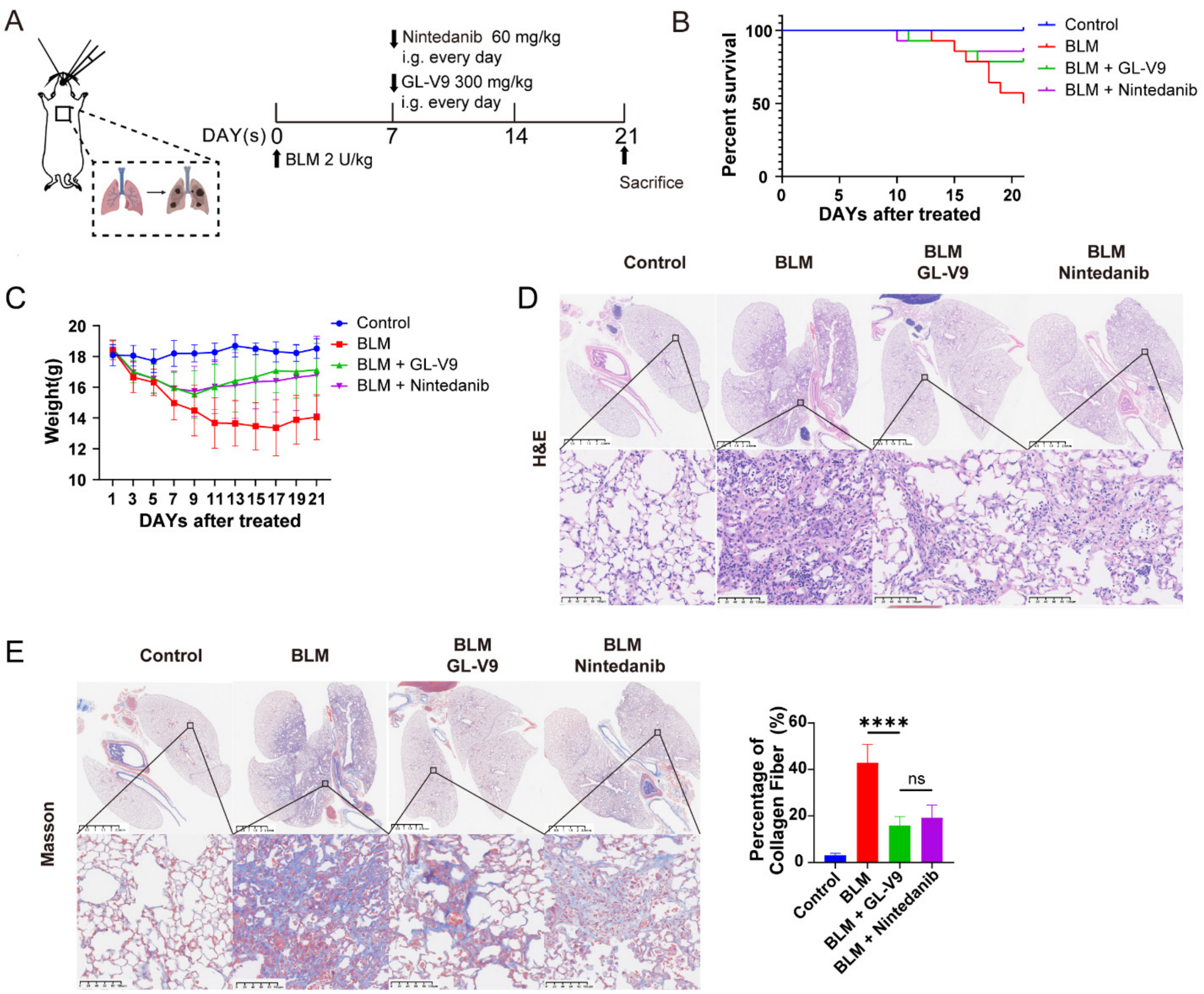

Figure S2 GL-V9 shows comparable therapeutic effects as Nintedanib in the attenuation of pulmonary fibrosis in mice induced by bleomycin. (A) Schematic representation of the experimental design for evaluating the effects of GL-V9 or Nintedanib on bleomycin-induced pulmonary fibrosis in mice. Pulmonary fibrosis was induced by transoral instillation of bleomycin at a dose of 2 U/kg. GL-V9 was administered at a dose of 300 mg/kg daily, starting on day 7 after bleomycin administration. Nintedanib was administered at a dose of 60 mg/kg daily, starting on day 7 after bleomycin administration. The study was conducted in four groups: the saline control group (Control, n=7), the bleomycin-induced pulmonary fibrosis model group (BLM, n=14), the group treated with GL-V9 (BLM + GL-V9, n=14), and the group treated with Nintedanib (BLM + Nintedanib, n=14). (B) Survival curve of mice. (C) Weight changes in mice. (D, E) H&E stains and Masson stains of mouse lung tissues. In Masson staining, collagen fibers appear blue-green, while the cytoplasm is stained red. The collagen fiber area in Masson-stained sections is quantified. Scale bar for the main image: 2.5 mm; enlarged section scale bars: 100 μm. Data are shown as mean ± SEM. ns $p > .05$, ** $p < .01$, *** $p < .001$, **** $p < .0001$.
