# Supplementary material for: Inhibition of Rho GEFs attenuates pulmonary fibrosis through suppressing myofibroblast activation and reprogramming profibrotic macrophages
Source: Cell Death Dis. 2025 Apr 11;16(1):278. doi: 10.1038/s41419-025-07573-5 (PMC11992128; doi:10.1038/s41419-025-07573-5)
Supplement: Supplementary file 3 — Supplementary Table [file 41419_2025_7573_MOESM3_ESM.pdf]

Supplementary Table

Table S1 Oligonucleotides used for qRT-PCR.

Gene Names	Primers	
ACTA2 (Mus)	sense	GTCCCAGACATCAGGGAGTAA
	antisense	TCGGATACTTCAGCGTCAGGA
FN1 (Mus)	sense	ATGTGGACCCCTCCTGATAGT
	antisense	GCCCAGTGATTTCAGCAAAGG
COL1A1 (Mus)	sense	GCTCCTCTTAGGGGCCACT
	antisense	CCACGTCTCACCATTGGGG
Mrc1 (Mus)	Sense	CTCTGTTCAGCTATTGGACGC
	antisense	CGGAATTTCTGGGATTCAGCTTC
Arg1 (Mus)	sense	CTCCAAGCCAAAGTCCTTAGAG
	antisense	AGGAGCTGTCATTAGGGACATC
TGFB1 (Mus)	sense	CTCCCGTGGCTTCTAGTGC
	antisense	GCCTTAGTTTGGACAGGATCTG
GAPDH (Mus)	sense	CTGGGCTACACTGAGCACC
	antisense	AAGTGGTCGTTGAGGGCAATG
