# Supplementary material for: Inhibition of Rho GEFs attenuates pulmonary fibrosis through suppressing myofibroblast activation and reprogramming profibrotic macrophages
Source: Cell Death Dis. 2025 Apr 11;16(1):278. doi: 10.1038/s41419-025-07573-5 (PMC11992128; doi:10.1038/s41419-025-07573-5)

Figure 3F

WT control

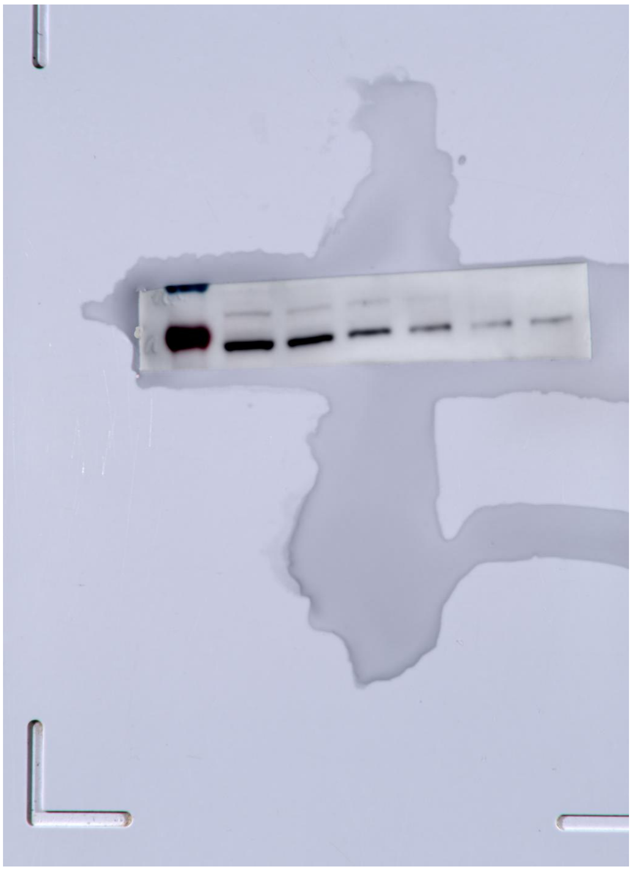

WT GL-V9

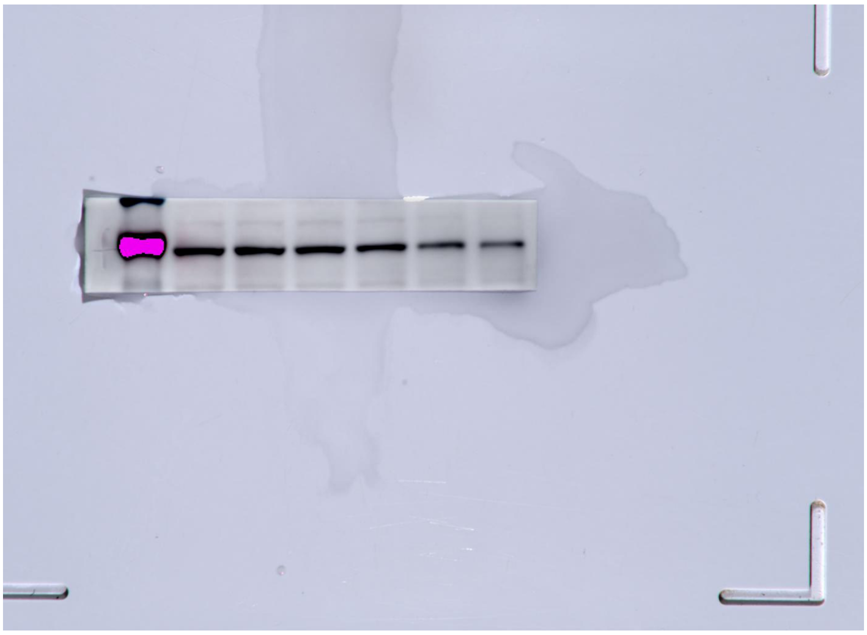

992R>A control

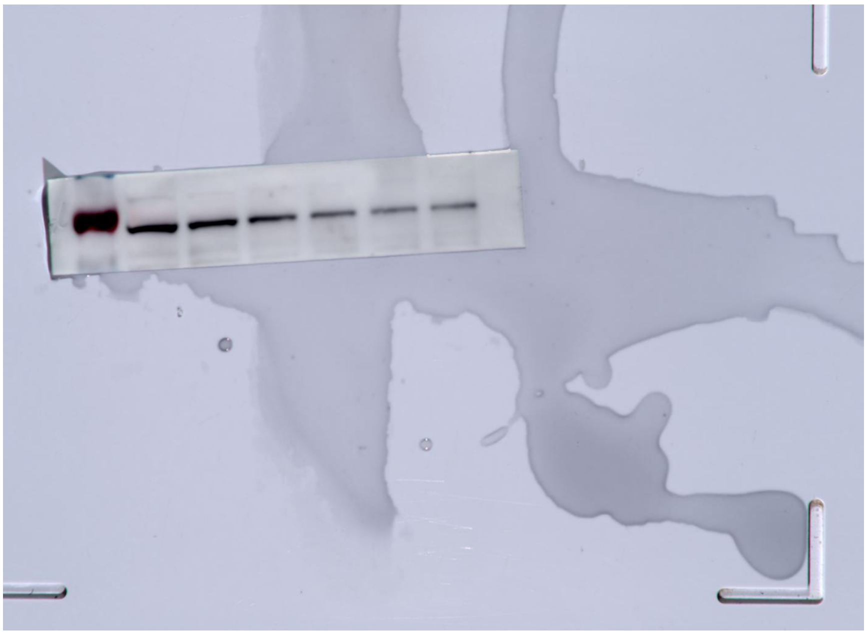

992R>A GL-V9

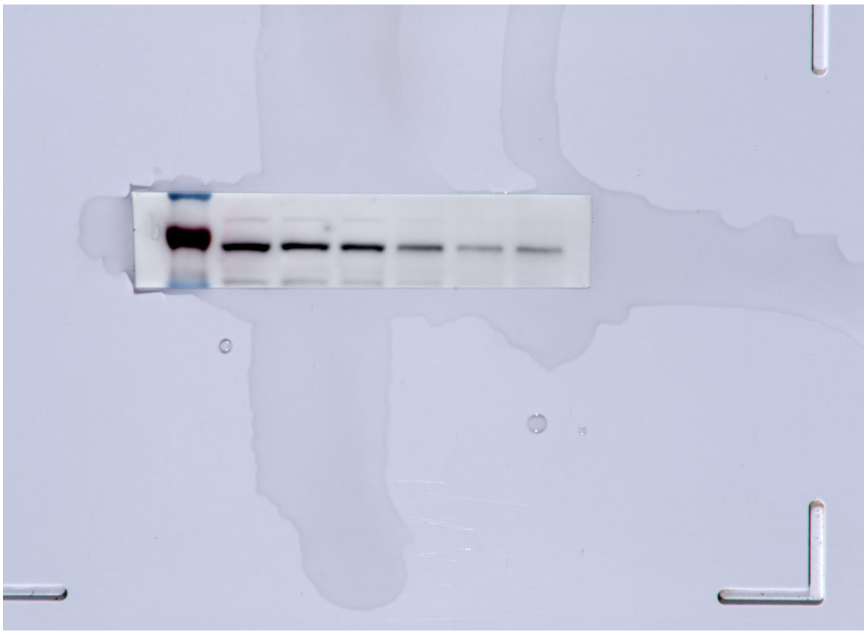

Figure 3G

Ha-p115 RhoGEF

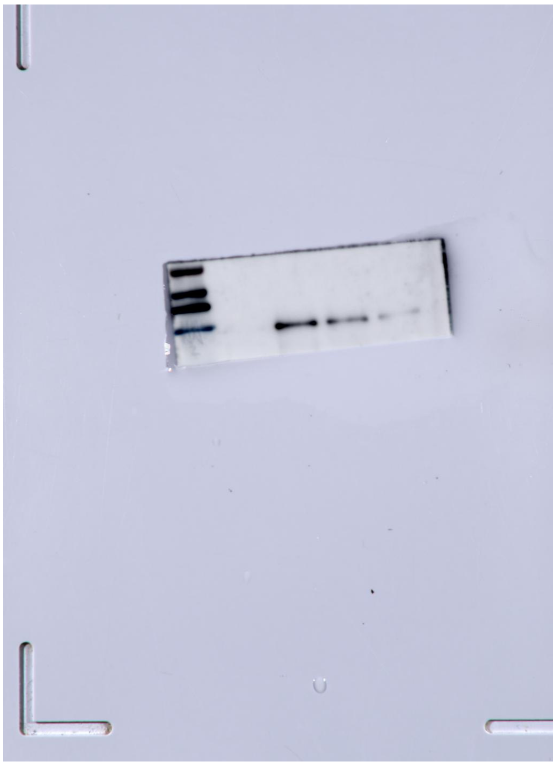

GST-RhoA

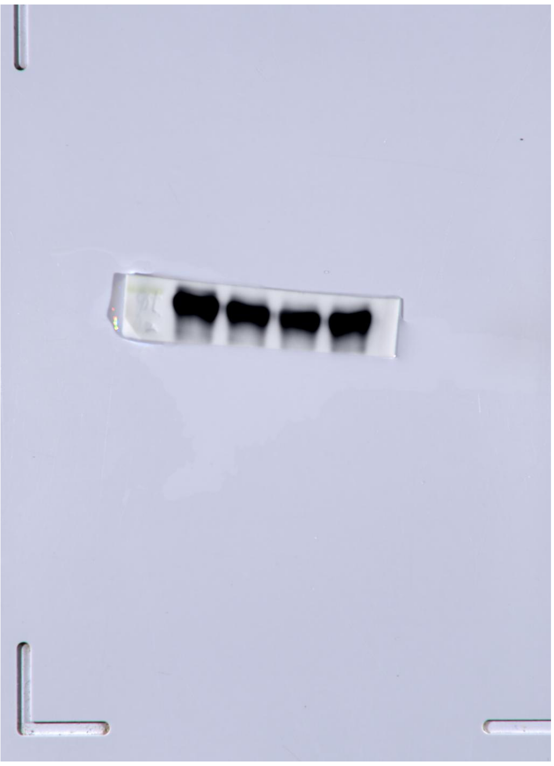

Figure 3H

HA-RhoA

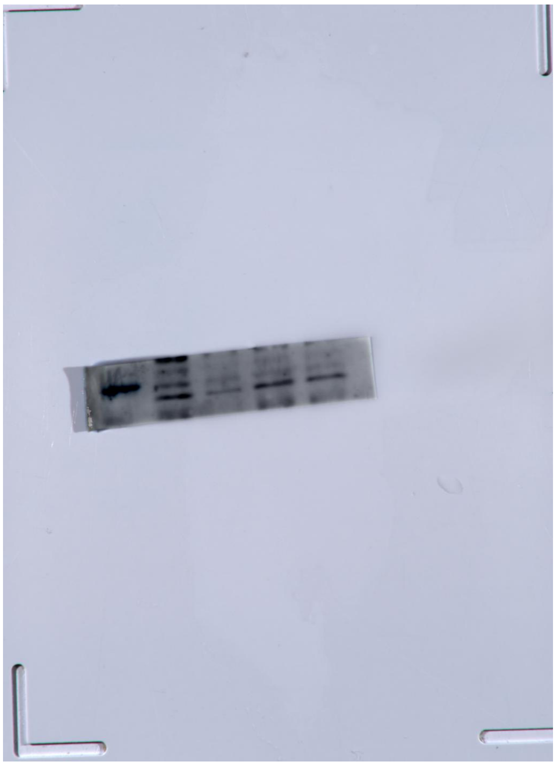

GST

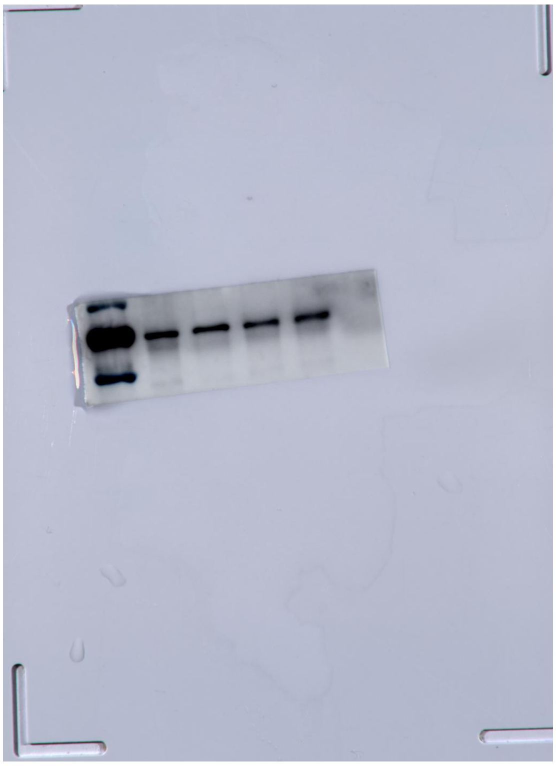

Figure 4A

RhoA-GTP

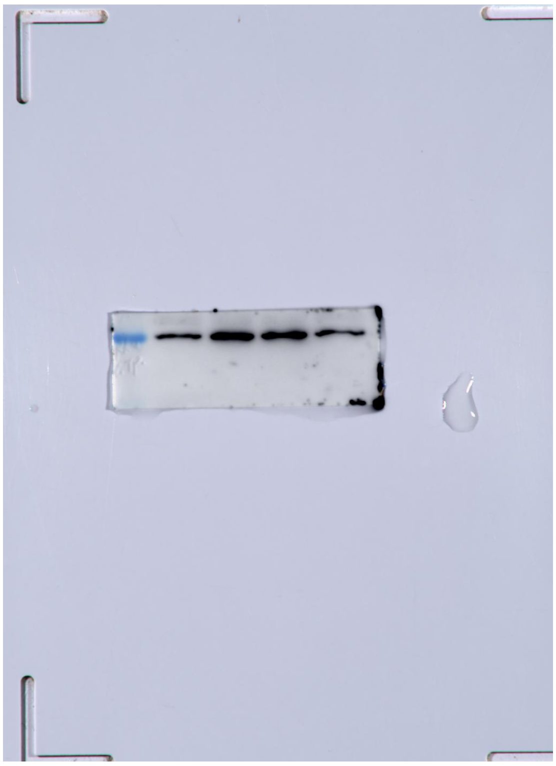

RhoA-GTotal

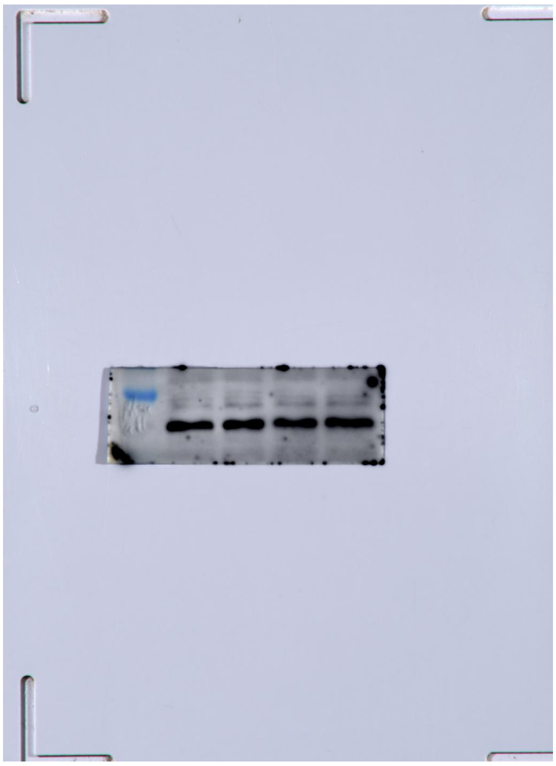

β-actin

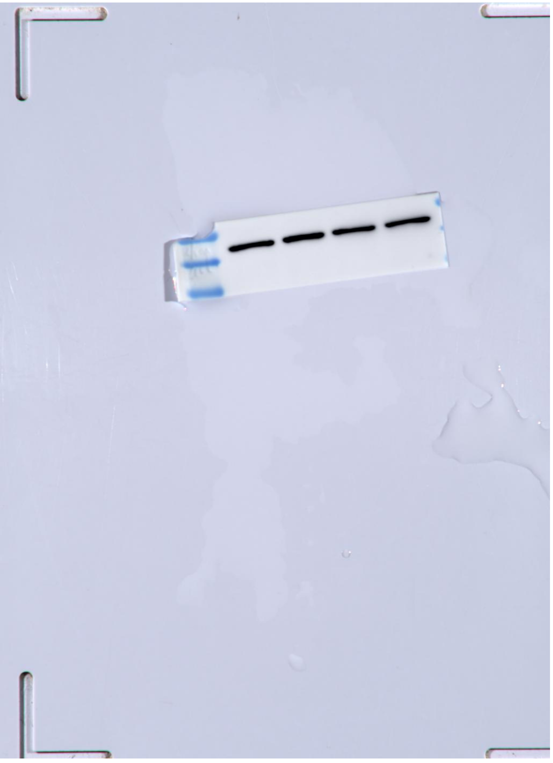

Figure 4E

p-MLC

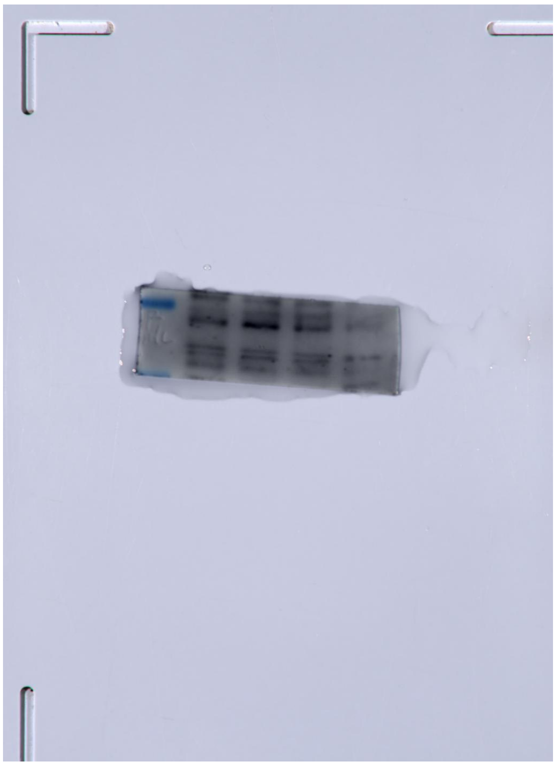

MLC

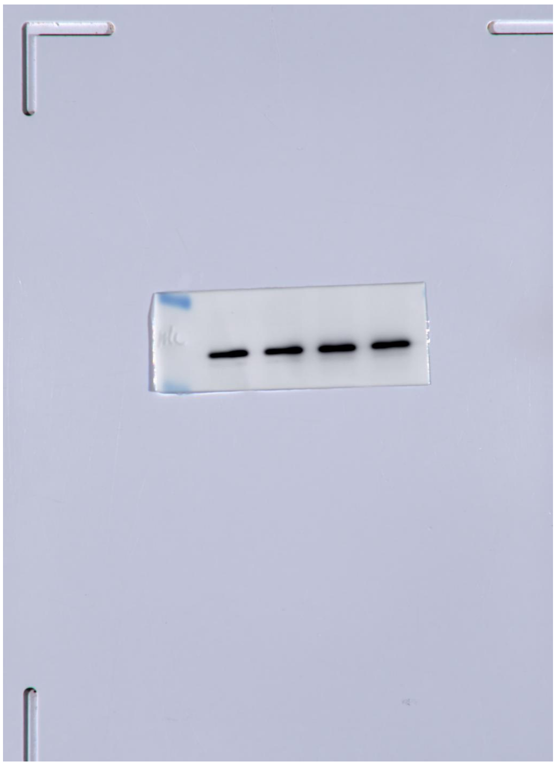

β-actin

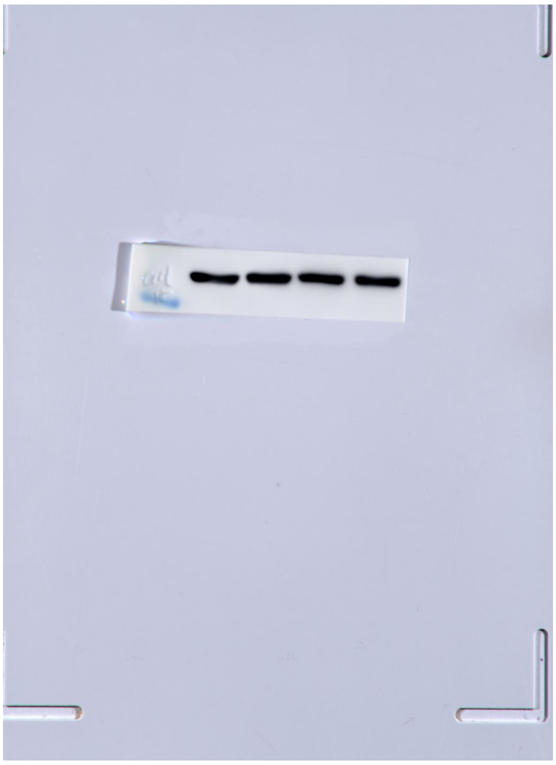

Figure 4I

p-MLC

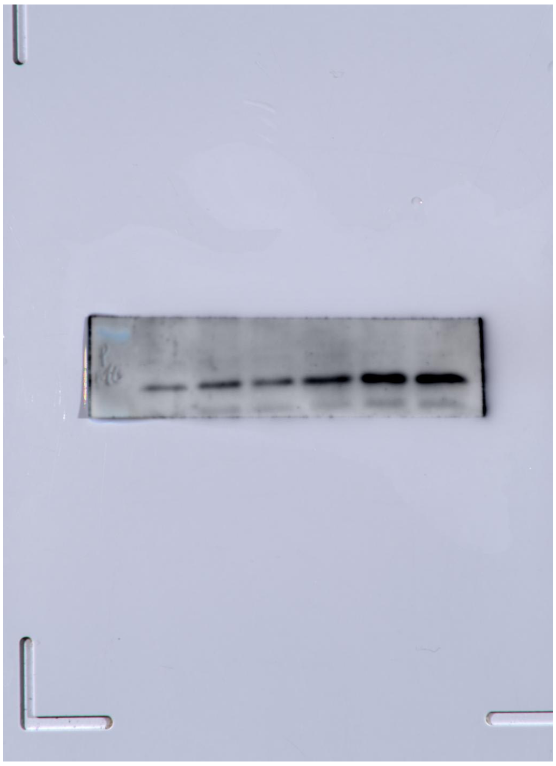

MLC

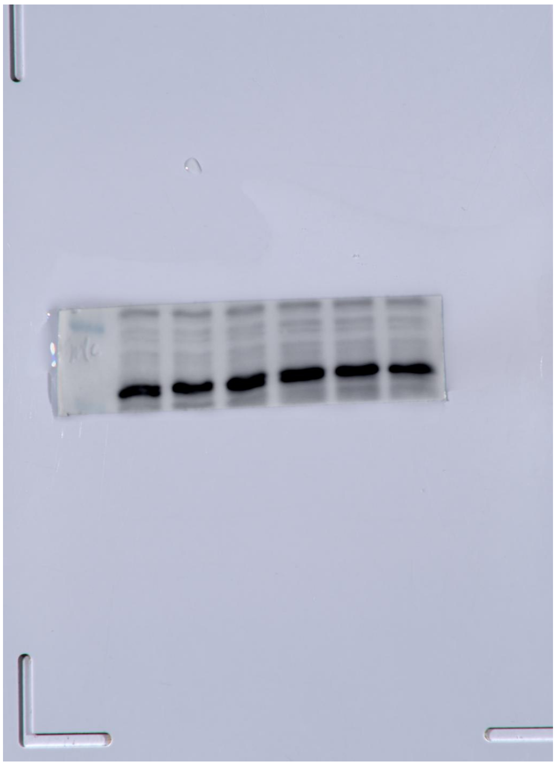

β-actin

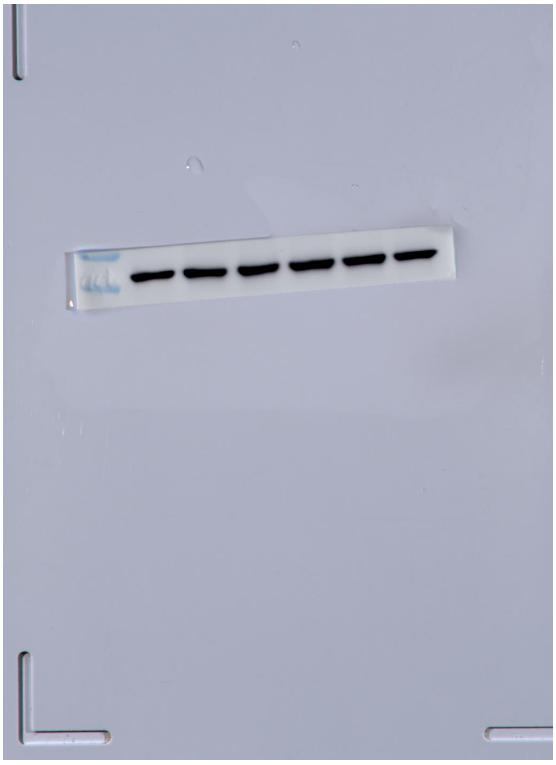

Figure 5A

Collagen I

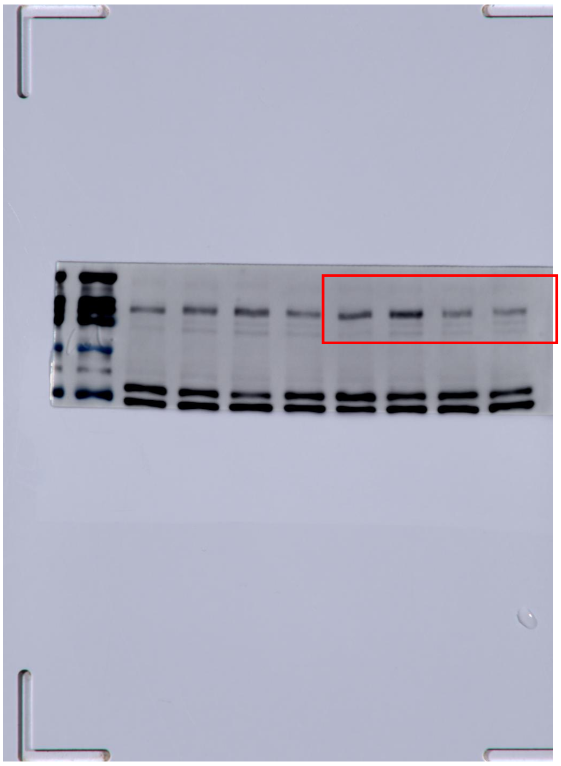

β-actin (Collagen I)

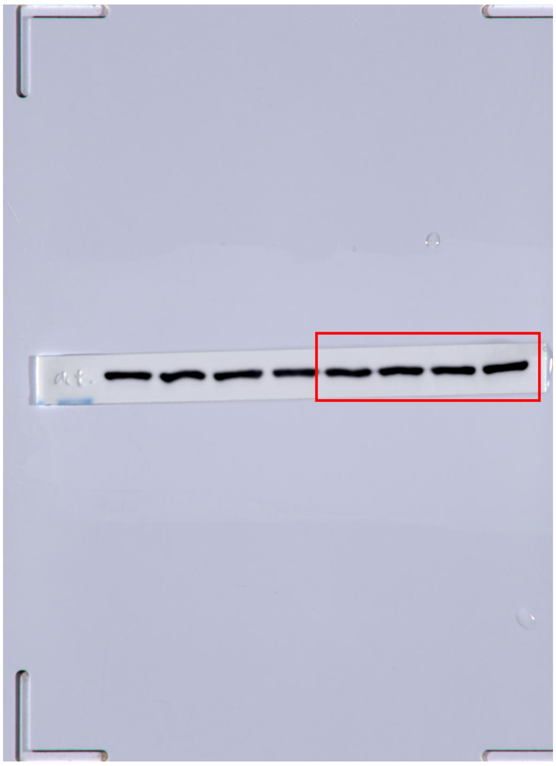

Fibronectin

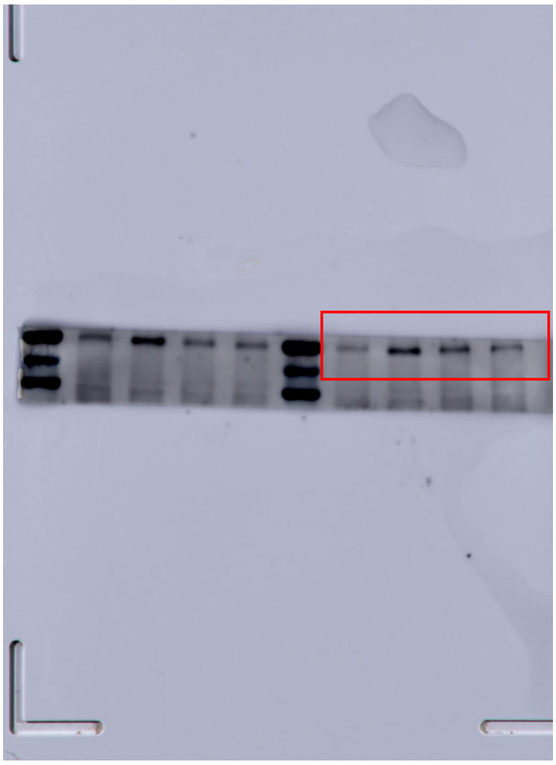

α-SMA

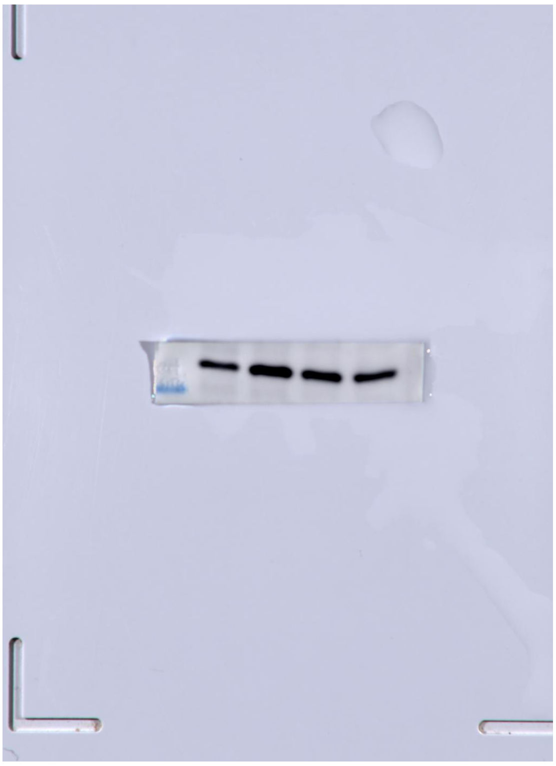

β-actin (Fibronectin and α-SMA)

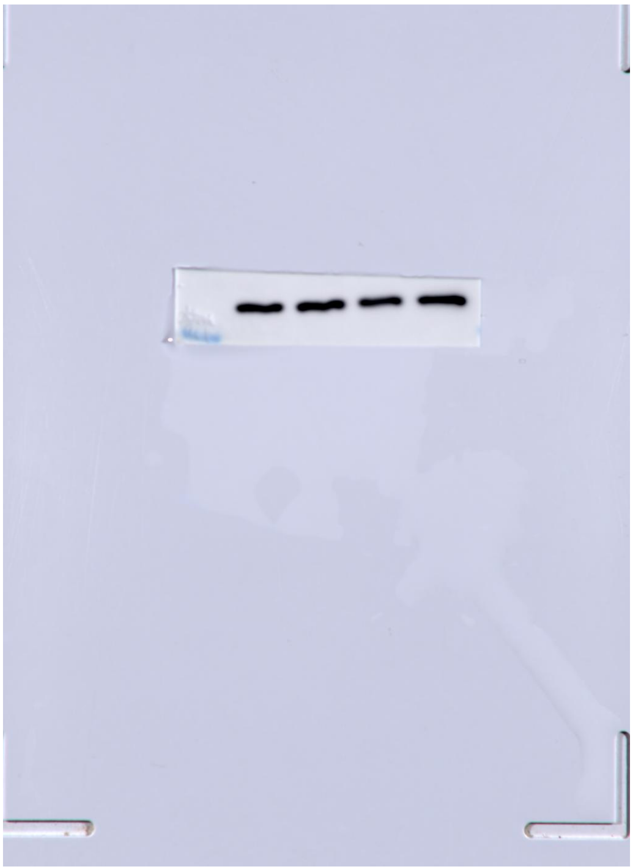

Figure 5D

Collagen I

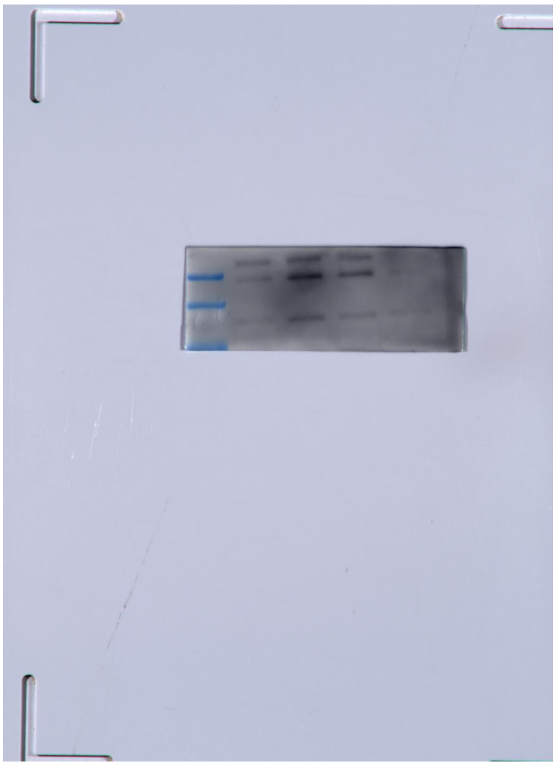

α-SMA

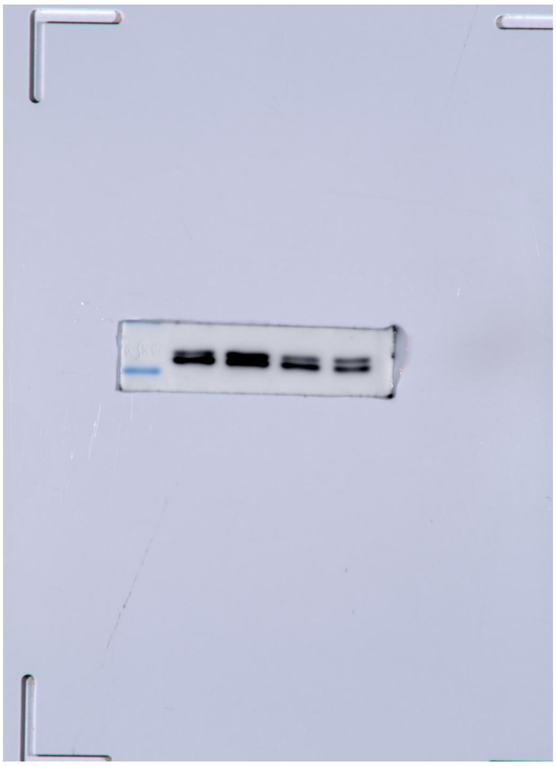

β-actin

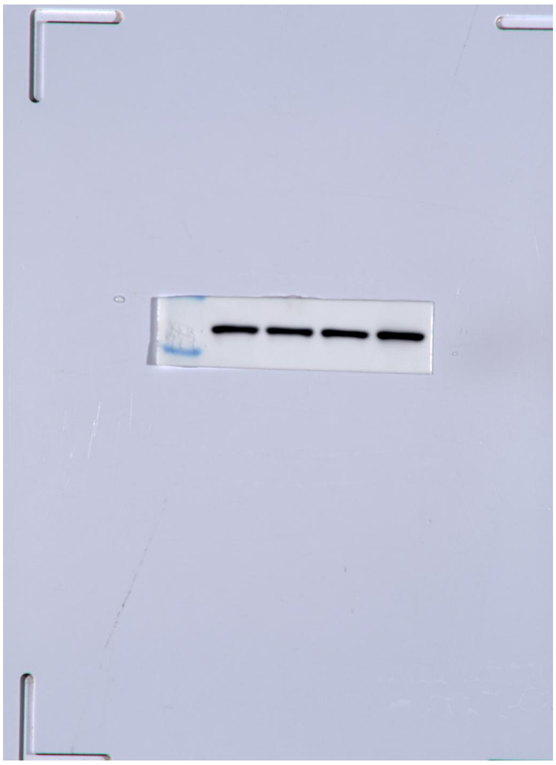

Figure 5G

MRTF-A

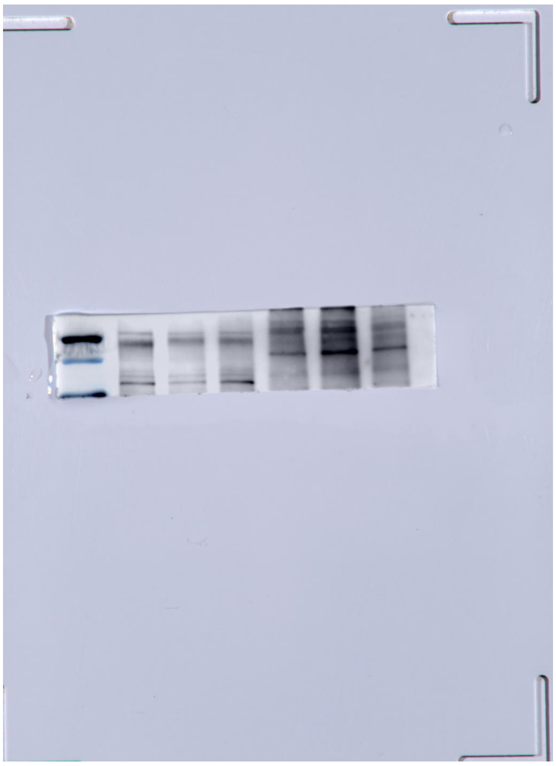

Lamin A

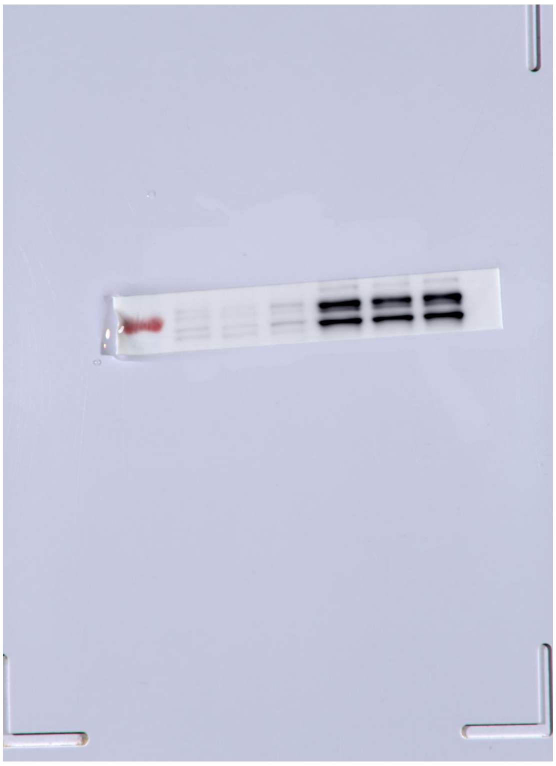

β-Tubulin

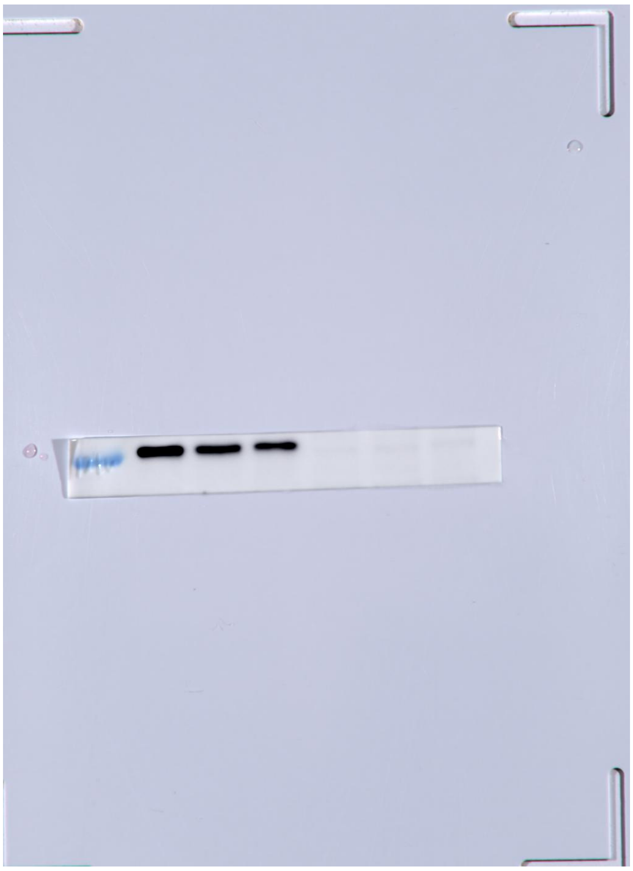

Figure 5J

p-LATS1

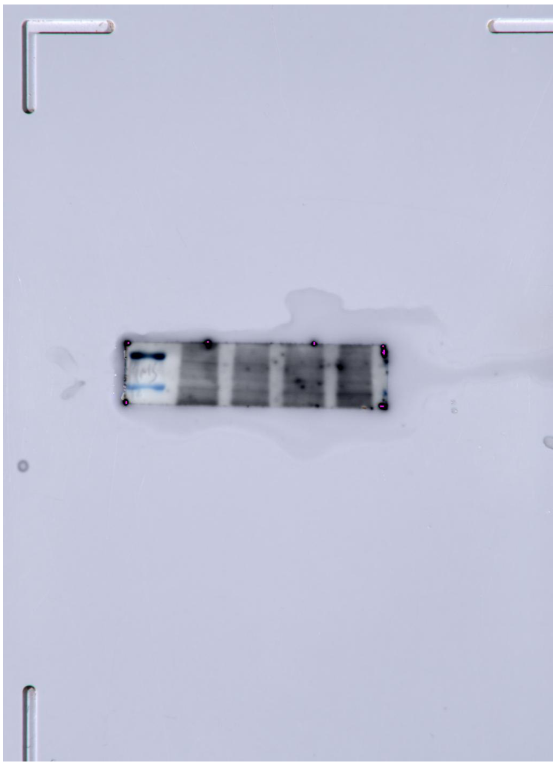

p-YAP

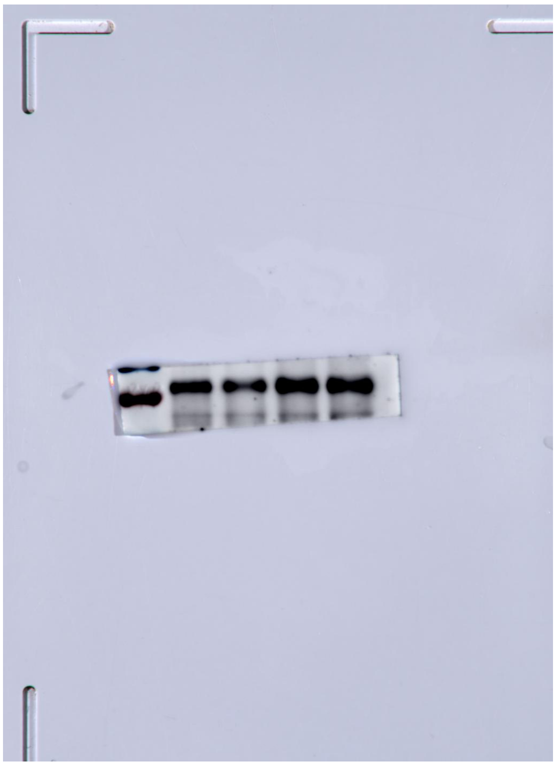

β-actin

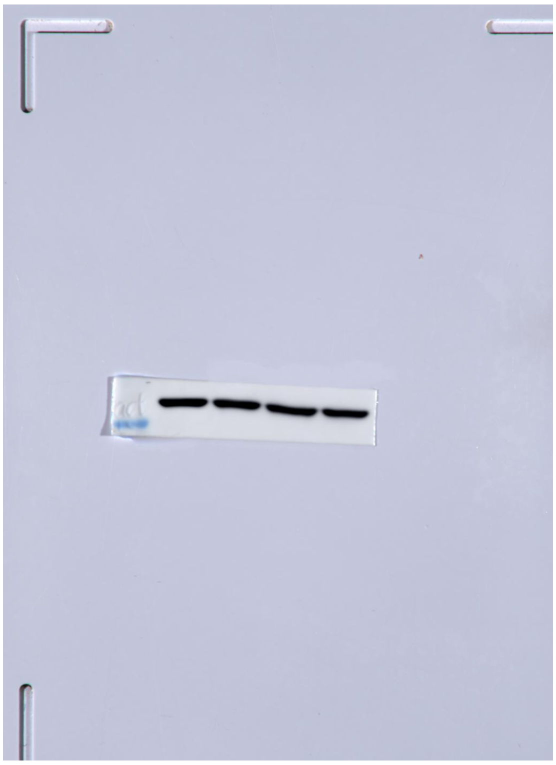

Figure 5K

YAP

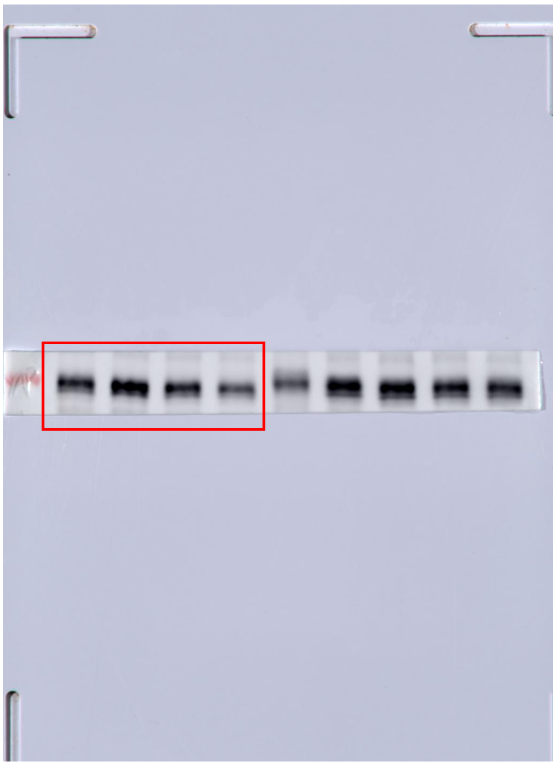

TAZ

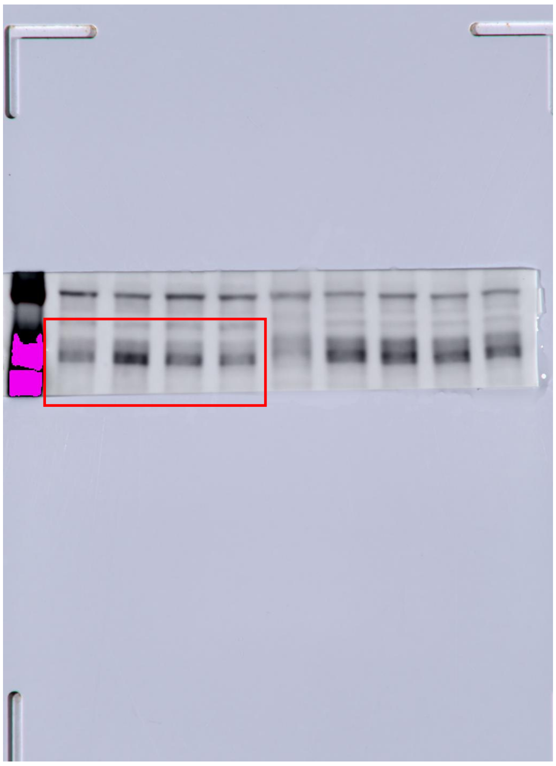

β-actin

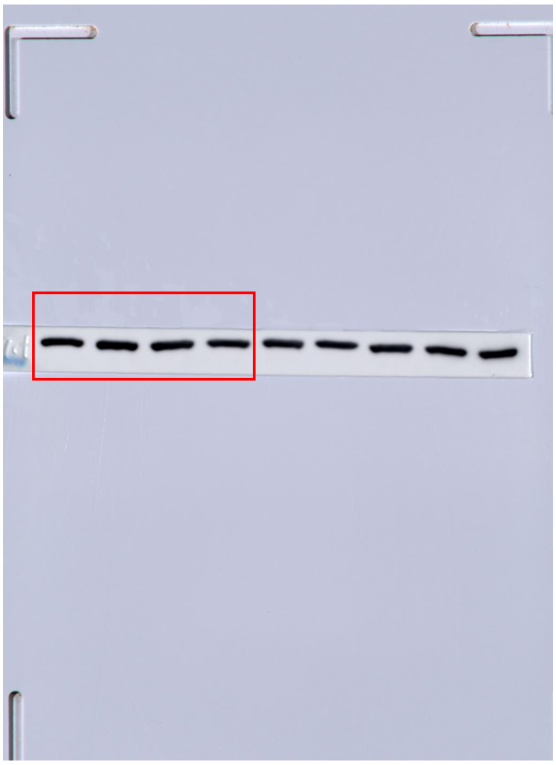

Figure 7C

CD206

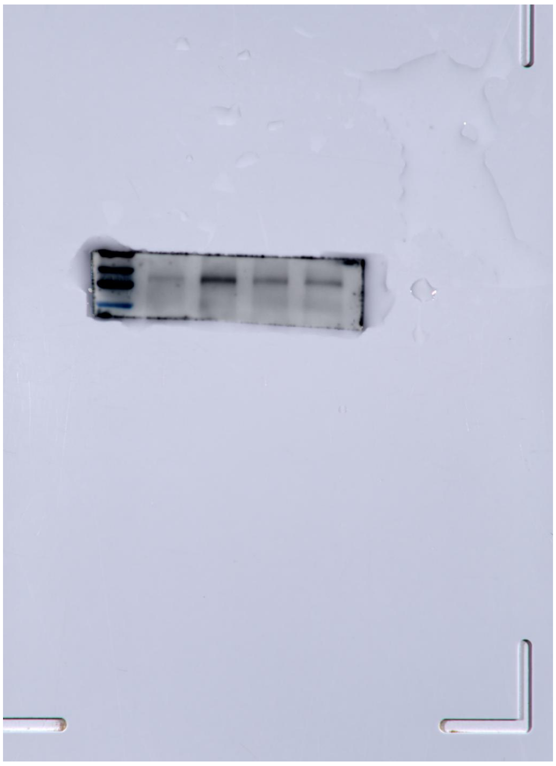

Arg1

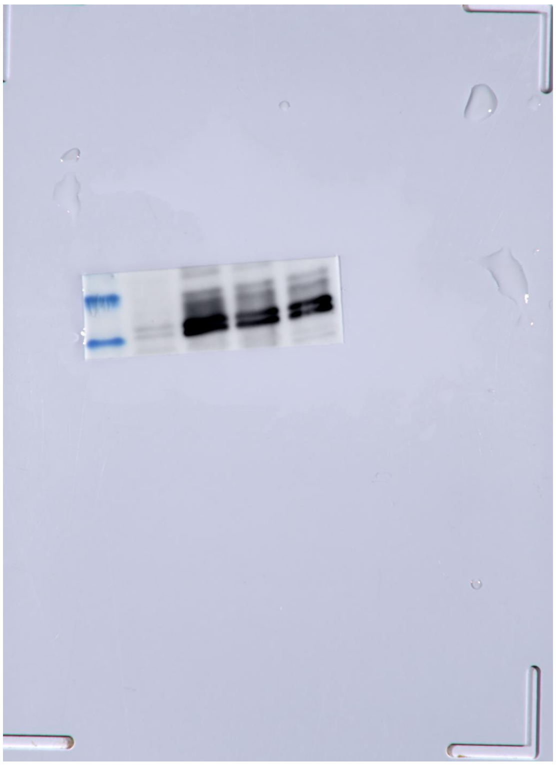

β-actin

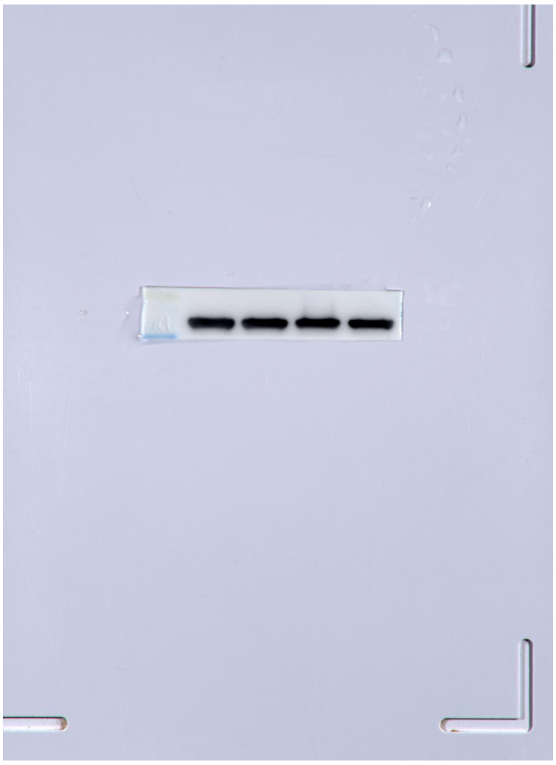

Figure 7D

CD206

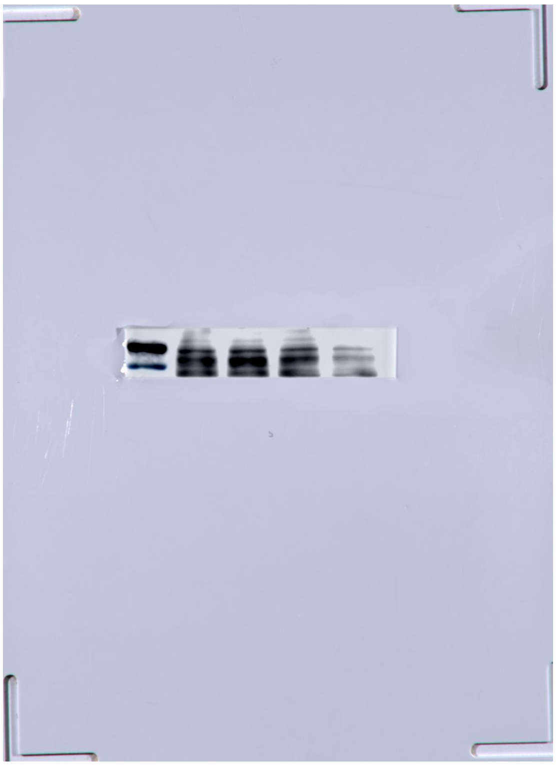

Arg1

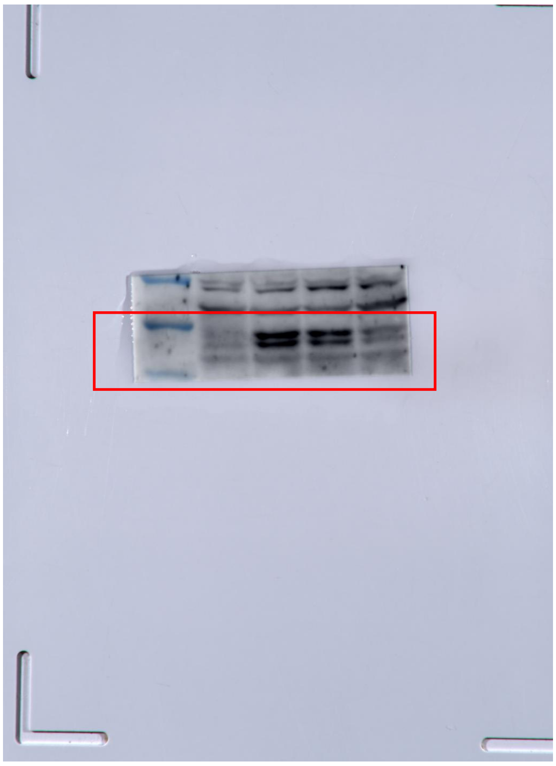

β-actin

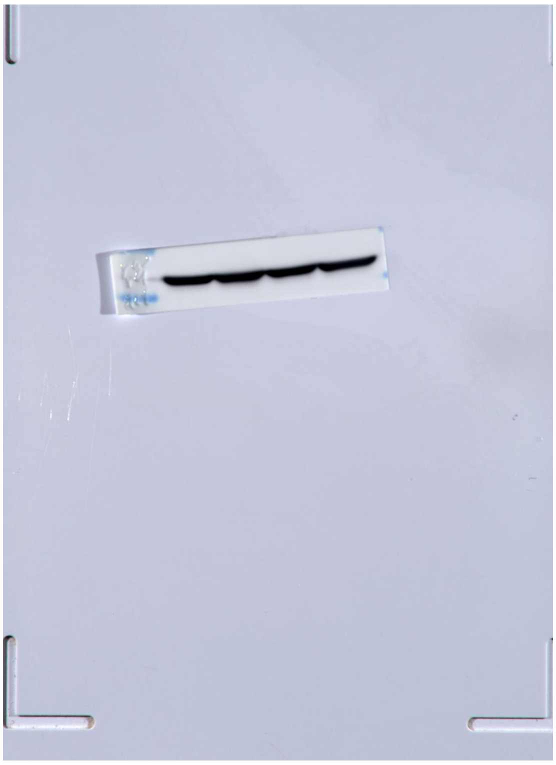

Figure 7F

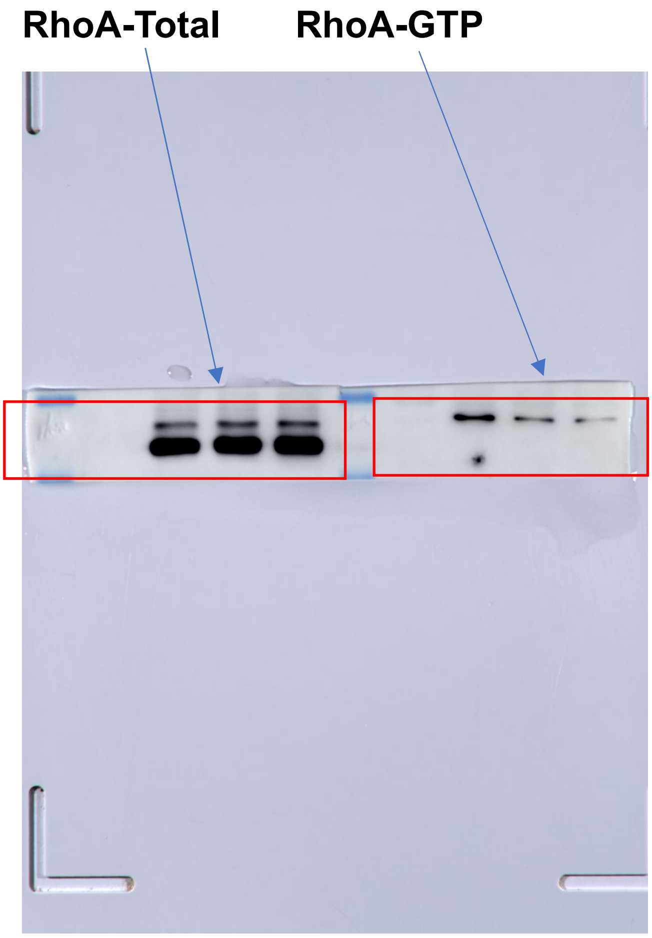

Figure 7G

p-MLC

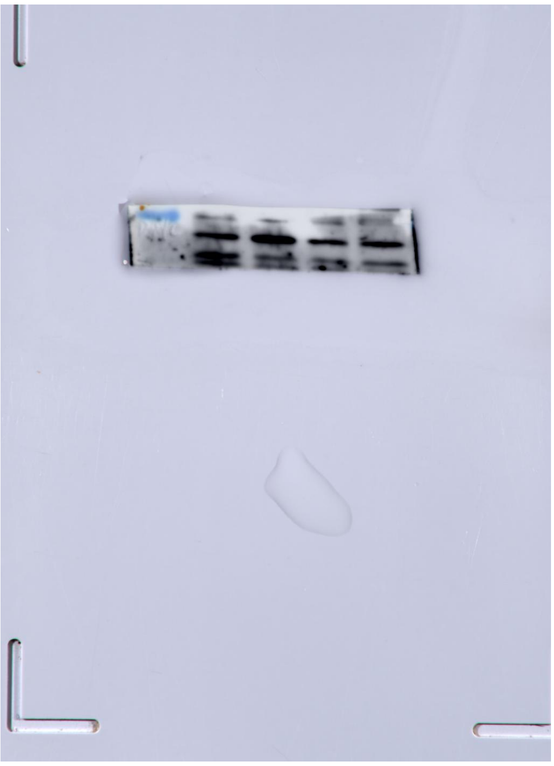

MLC

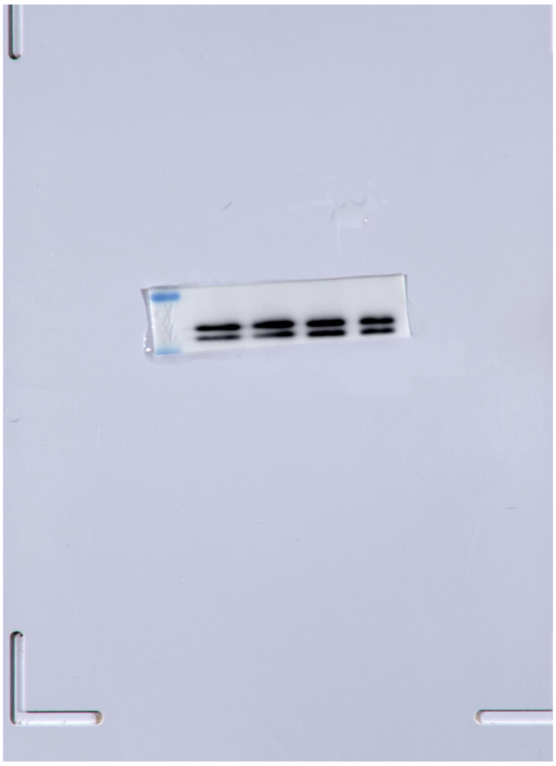

β-actin

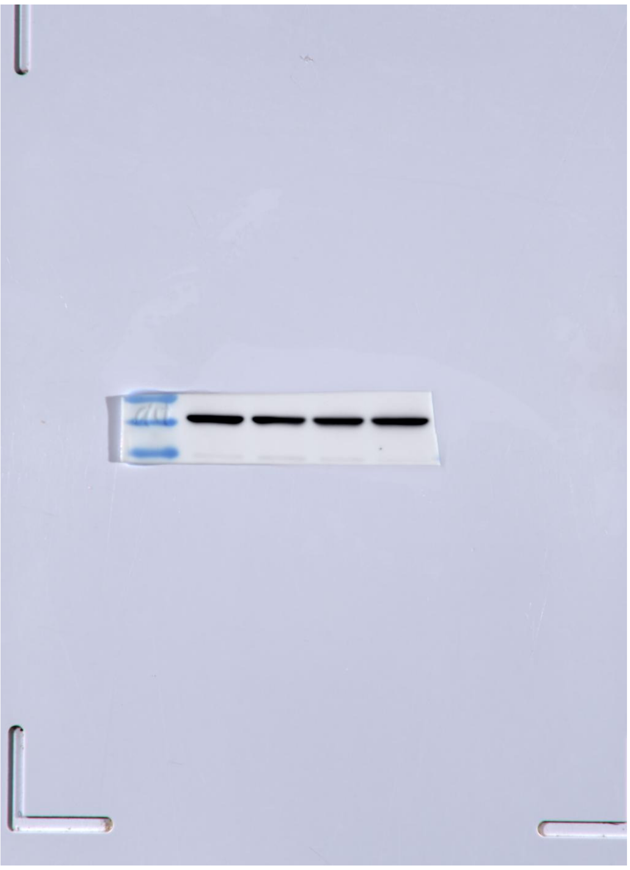

Figure 7J

Arg1

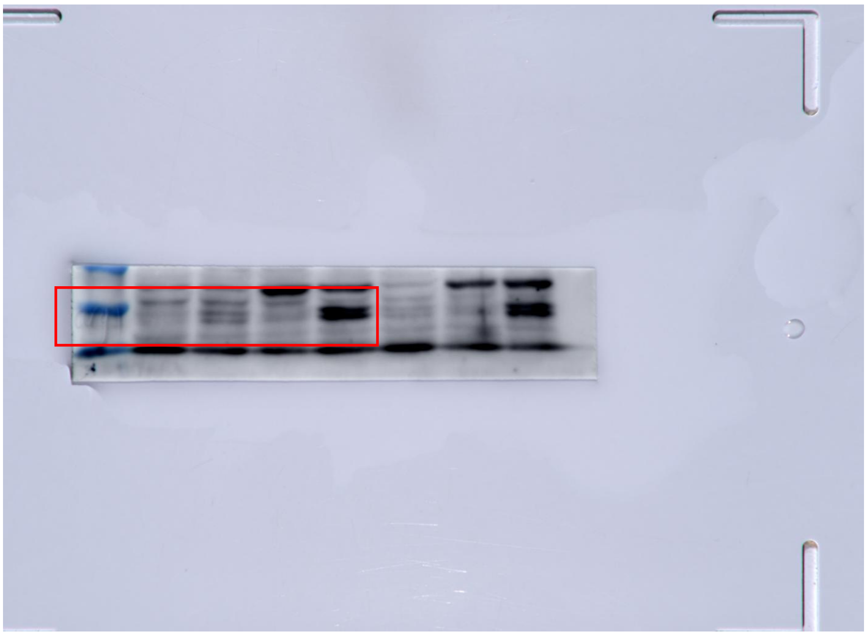

p-STAT3

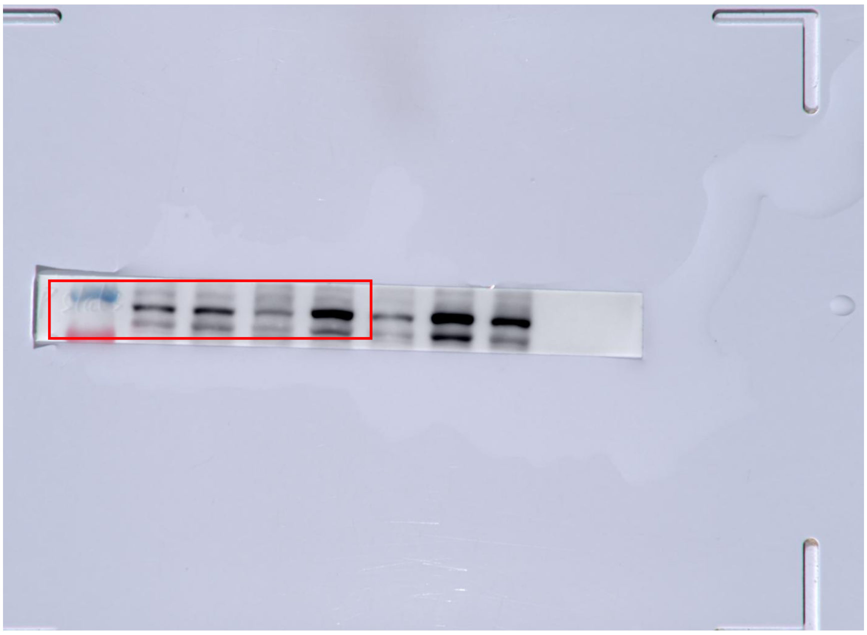

β-actin (Arg1 and p-STAT3)

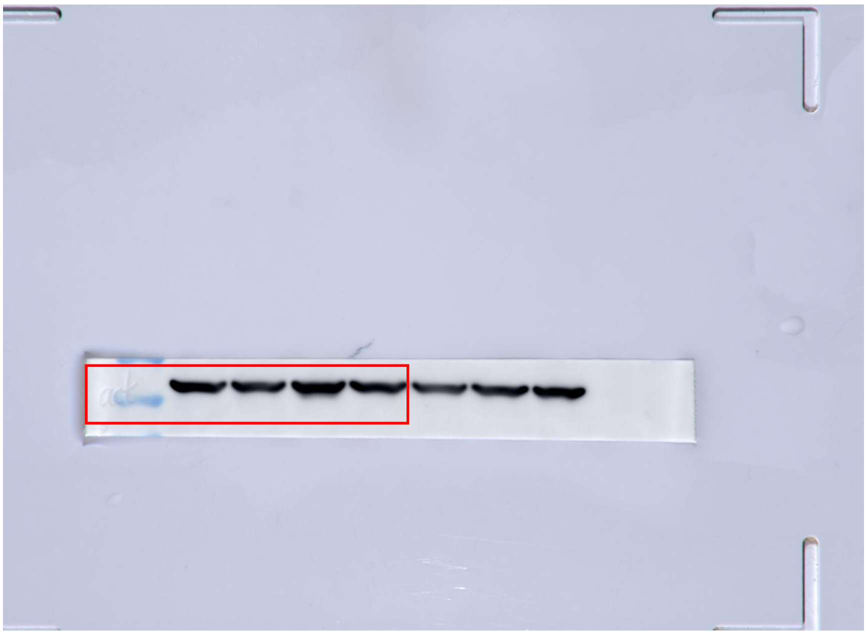

p-STAT6

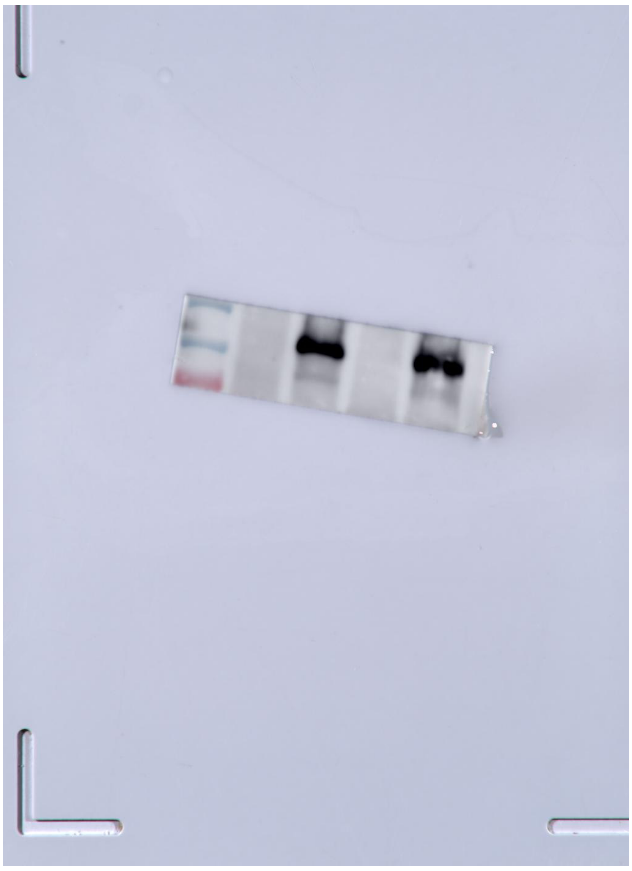

β-actin (p-STAT6)

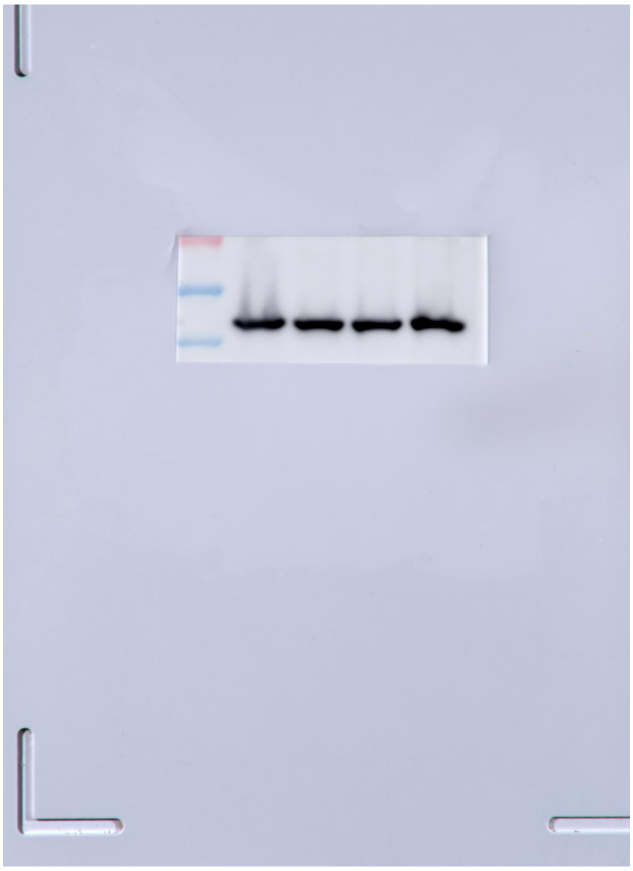

Figure 7L

p-STAT3 (BMDM)

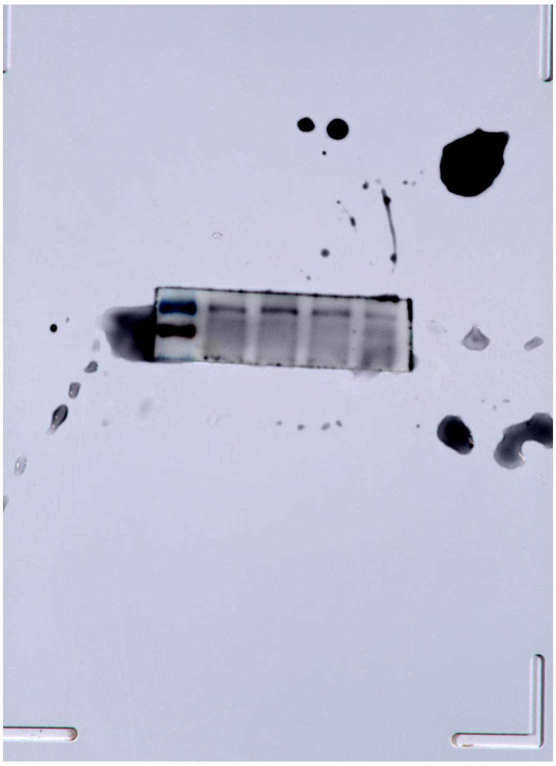

β-actin (BMDM)

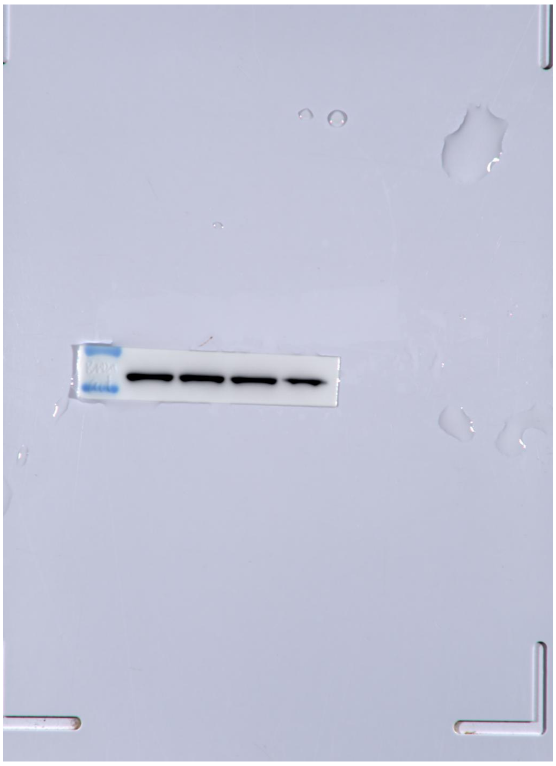

p-STAT3 (RAW)

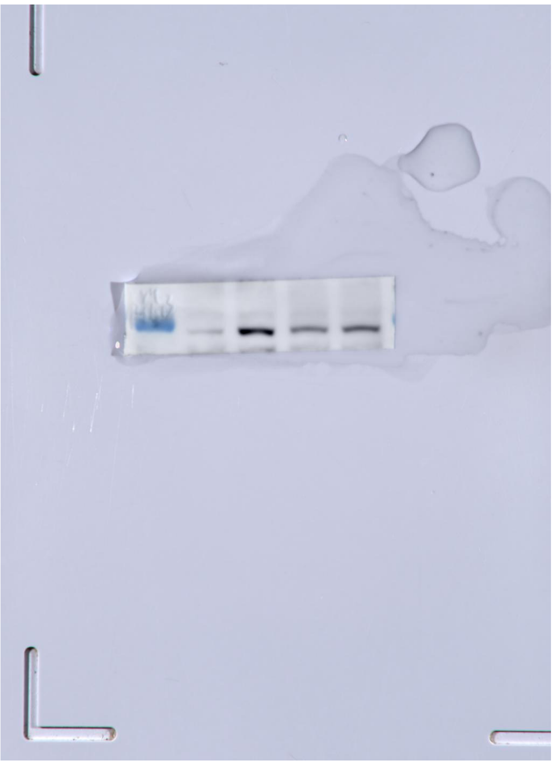

β-actin (RAW)

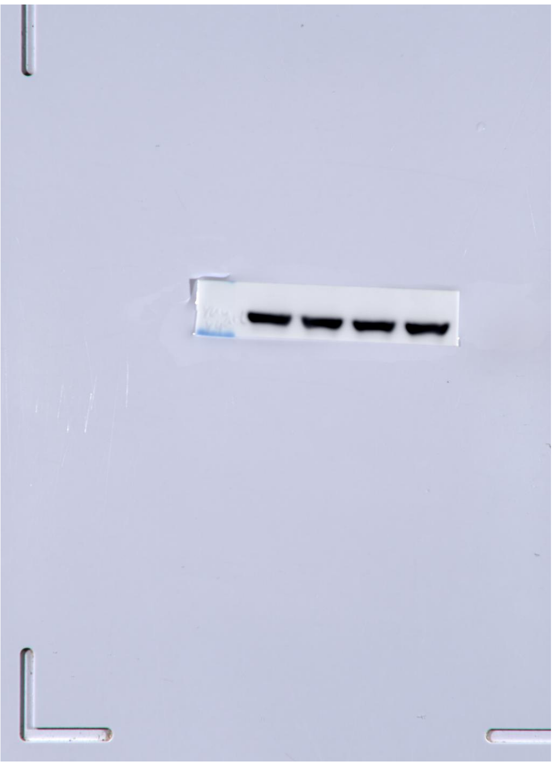

Figure 7O

Arg1

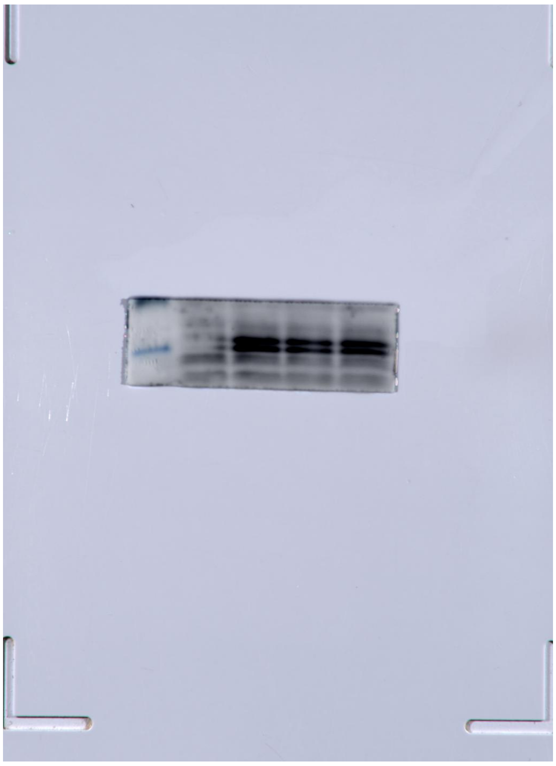

p-STAT3

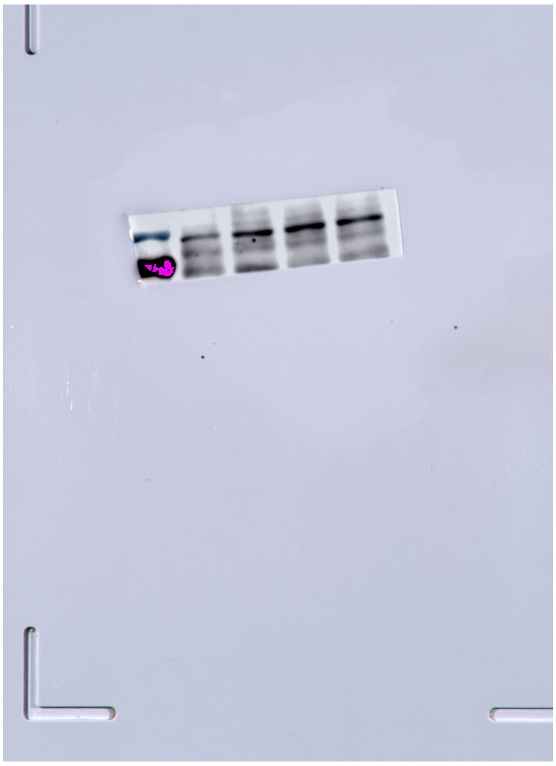

β-actin

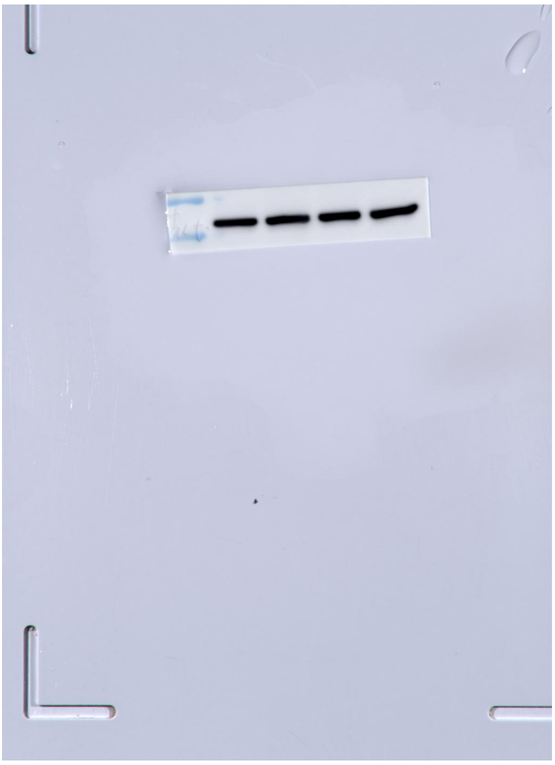

Supplement: Supplementary file 4 — Original Data [file 41419_2025_7573_MOESM4_ESM.pdf]
